# Digital Health Interventions for Adult Patients With Cancer Evaluated in Randomized Controlled Trials: Scoping Review

**DOI:** 10.2196/38333

**Published:** 2023-01-06

**Authors:** Kyunghwa Lee, Sanghee Kim, Soo Hyun Kim, Sung-Hee Yoo, Ji Hyun Sung, Eui Geum Oh, Nawon Kim, Jiyeon Lee

**Affiliations:** 1 College of Nursing Konyang University Daejeon Republic of Korea; 2 College of Nursing and Mo-im Kim Nursing Research Institute, Yonsei Evidence Based Nursing Center of Korea: Affiliation of the Joanna Briggs Institution Yonsei University Seoul Republic of Korea; 3 Department of Nursing Inha University Inchon Republic of Korea; 4 College of Nursing Chonnam National University Gwangju Republic of Korea; 5 College of Nursing Kosin University Busan Republic of Korea; 6 Yonsei Medical Library Yonsei University Seoul Republic of Korea

**Keywords:** digital health, adult, neoplasms, randomized controlled trial, mobile phone

## Abstract

**Background:**

Digital care has become an essential component of health care. Interventions for patients with cancer need to be effective and safe, and digital health interventions must adhere to the same requirements.

**Objective:**

The purpose of this study was to identify currently available digital health interventions developed and evaluated in randomized controlled trials (RCTs) targeting adult patients with cancer.

**Methods:**

A scoping review using the JBI methodology was conducted. The participants were adult patients with cancer, and the concept was digital health interventions. The context was open, and sources were limited to RCT effectiveness studies. The *PubMed, CINAHL, Embase, Cochrane Library, Research Information Sharing Service*, and *KoreaMed* databases were searched. Data were extracted and analyzed to achieve summarized results about the participants, types, functions, and outcomes of digital health interventions.

**Results:**

A total of 231 studies were reviewed. Digital health interventions were used mostly at home (187/231, 81%), and the *web-based* intervention was the most frequently used intervention modality (116/231, 50.2%). Interventions consisting of *multiple* functional components were most frequently identified (69/231, 29.9%), followed by those with the *self-manage* function (67/231, 29%). *Web-based* interventions targeting symptoms with the *self-manage* and *multiple* functions and *web-based* interventions to *treat* cognitive function and fear of cancer recurrence consistently achieved positive outcomes. More studies supported the positive effects of *web-based* interventions to *inform* decision-making and knowledge. The effectiveness of digital health interventions targeting anxiety, depression, distress, fatigue, health-related quality of life or quality of life, pain, physical activity, and sleep was subject to their type and function. A relatively small number of digital health interventions specifically targeted older adults (6/231, 2.6%) or patients with advanced or metastatic cancer (22/231, 9.5%).

**Conclusions:**

This scoping review summarized digital health interventions developed and evaluated in RCTs involving adult patients with cancer. Systematic reviews of the identified digital interventions are strongly recommended to integrate digital health interventions into clinical practice. The identified gaps in digital health interventions for cancer care need to be reflected in future digital health research.

## Introduction

### Background

Digital health care has become a necessity in delivering care. Digital health is defined as health services and information to manage illness and health risks delivered or enhanced through the internet and related technologies, that is, information and communication technology [1,2]. The term digital health is rooted in eHealth [1], which encompasses mobile health, telemedicine, telemonitoring, digital therapeutics, digital health analytics, and digital health systems [3,4]. Wearables, mobile apps, and web pages are examples of digital technologies that are applied to enhance health care.

Digital health receives increased attention because it is expected to improve access to health care, reduce inefficiencies in the health care system, improve quality of care, and lower health care costs [2]. The World Health Organization has recognized the value of digital technologies in advancing universal health coverage and provides recommendations for their use [5]. The COVID-19–related restrictions, which limit person-to-person contact, have accelerated the development and implementation of digital health in practice, potentially making it possible to continue providing the best possible care. Promising outcomes have been suggested from digital health intervention trials; for example, Basch et al [6] incorporated electronic patient-reported outcome monitoring of 12 symptoms during routine cancer treatment and demonstrated less decline in health-related quality of life (HRQoL), less frequent readmission, longer continuation of chemotherapy treatment, and longer quality-adjusted survival.

However, current systematic and scoping reviews on digital health interventions for patients with cancer provide information that is too fragmentary to enable a comprehensive understanding of available digital health interventions for cancer care. Reviews were often focused on specific cancer populations. The most frequently studied were adolescents and young adults, considering their familiarity with digital solutions [7-12]. Mixed outcomes were identified for symptom management [8], physical symptoms and functioning, emotional distress, health behaviors, neurocognitive functioning, health knowledge, and self-efficacy of adolescents and young adults [11]. Digital self-management was not effective in improving HRQoL or moderate-to-vigorous physical activity [9]. Some studies have reviewed the application of digital health for specific types of cancers, that is, breast cancer [13], prostate cancer [14], melanoma [15], and hematologic malignancy [16]. Although digital health is applicable across the cancer continuum, reviews were limited to the postoperative phase [13], survivorship [17,18], and palliative care [7,19,20]. There exists a single digital health review that spans across pediatric patients’ cancer continuum (from treatment to survivorship) [11].

A limited number of reviews exist about the effectiveness of particular digital health intervention modalities targeting specific outcomes: mobile health is ineffective for oral anticancer drug adherence [21], telemedicine demonstrates similar or improved care in patients with hematologic malignancies [16], and activity trackers improve activity level and HRQoL [22]. In terms of the outcomes of digital health interventions, improvements in physical activity or exercise [23] and patient-provider communication [24]; benefits in symptom assessment and management, functional capacity, and HRQoL [3,25]; decreases in emergency room visits, unplanned hospitalization, and hospital days; and increased survival [3] have been supported. The use of digital health to support survivorship care planning resulted in positive physical and psychosocial effects, whereas there were mixed effects for fear and anxiety [18]. Inconsistent outcomes have been achieved for diet among cancer survivors [23] and for psychological outcomes of patients with cancer in the treatment or survivorship phase [26] and those receiving palliative care [7].

Digital health care for patients with cancer needs to be effective and safe [27]. A comprehensive overview of available digital health interventions for patients with cancer will help us to understand the current status of digital health in cancer care and identify gaps and areas for further development while readying for the inevitable surge in digital technologies.

### Objectives

The purpose of this scoping review was to identify currently available digital health interventions developed and evaluated in randomized controlled trials (RCTs) among adult patients with cancer. The primary research question was as follows: *What digital health interventions were developed and evaluated for adult patients with cancer?*

The subquestions were as follows:

1. What were the characteristics of targets for digital health interventions (age, sex, type and stage of cancer, and trajectory)?
2. What were the types and functions of digital health interventions?
3. How were the digital health interventions applied (frequency, duration, and time spent for intervention)?
4. What nursing problems were sought for intervention with digital health interventions?
5. What were the outcomes of digital health interventions?

## Methods

### Design

A scoping review was conducted in accordance with the JBI methodology [28]. The population, concept, and context framework for this scoping review was as follows: (1) population: adult patients with cancer or cancer survivors, (2) concept: digital health interventions, and (3) context: open (no limitation).

### Search Strategy

The search strategy aimed to identify published RCTs that reported the efficacy of digital health interventions among adult patients with cancer. An initial limited search of *PubMed* was undertaken to identify articles on the topic. The words contained in the titles and abstracts of relevant articles and the index terms used to describe the articles were used to develop a full search strategy for *PubMed, CINAHL, Embase, Cochrane Library, Research Information Sharing Service,* and *KoreaMed.* Key search terms included all identified keywords, and index terms were adapted for each included database and information source (Multimedia Appendix 1). The reference lists of related sources of evidence were screened for additional studies.

### Study and Source of Evidence Selection

Studies published from 1999, considering the time when the definition of eHealth was first introduced [29], to July 26, 2021, and published in English and Korean were included. As this scoping review aimed to include outcomes from RCTs, the type of source was set as a published RCT reporting the effect of a digital health intervention; thus, unpublished studies or gray literature were excluded.

After the search, all identified citations were collated into *EndNote* (version 20.0; Clarivate), and duplicates were removed. The team of reviewers paired into 3 groups (SK and KL, JL and JHS, and SHK and S-HY) for screening and data extraction, considering that >5000 articles were retrieved through the database search.

To identify outcomes from digital health interventions applied to patients with cancer, studies that evaluated the effect of digital health interventions in the form of RCTs were included in the review. Trial runs of article selection and relevant discussions facilitated modification of the initial set of eligibility criteria. The final eligibility criteria were as follows: (1) participants needed to be adult patients with cancer (aged ≥18 years), but studies involving adolescents and young adults that included adult patients were also included in this review; and (2) interventions needed to demonstrate their effects in patients with cancer; thus, studies investigating interventions for patients with chronic disease in which patients with cancer also took part or interventions to promote cancer screening among healthy individuals were excluded.

Classic telephone service or replication of clinical service by substituting it using the telephone was not considered a digital intervention according to the criteria outlined by Marthick et al [25], who define the functions of digital health technologies as monitoring, tracking, and providing information on health as well as enabling communication. However, studies of automated systems (interactive voice response) were included if data were obtained from patients and patient-tailored feedback from professionals (tailored automated voice response) was delivered. Interventions in the form of simple videos contained in CD-ROMs or tablet devices were excluded. If the study assessed outcomes by applying digital assessment tools but the intervention itself was not considered digital, it was excluded.

Only RCTs that reported outcomes of the digital intervention were eligible to be included. In the case of pilot RCTs, if the study reported the effect of digital interventions, it was included for review. Cost-effectiveness was considered an effect of the intervention, whereas satisfaction was not.

A pilot selection using 25 randomly selected studies demonstrated 80% agreement among all 6 reviewers in selecting the source of evidence based on titles and abstracts. Among the initially selected 5164 articles, potentially relevant sources were retrieved, and the full text of selected citations was assessed in detail against the eligibility criteria by 2 independent reviewers. Reasons for exclusion of sources of evidence after full-text review were recorded. Any disagreements that arose between the reviewers at each stage of the selection process were resolved through discussion with a third reviewer.

### Data Extraction

Data were extracted by 3 groups of 2 independent reviewers (a total of 6 reviewers) using a data extraction tool developed by the reviewers. The data extracted included specific details about the selected article (authors, year, title, and country in which the study was conducted), participants (age, sex, type of cancer, and phase of cancer journey), concept (types and functions of the digital health interventions), context (setting for the intervention), study methods (number of participants; details, frequency, and duration of the intervention; time spent administering the intervention; and types and details of controls) and key findings relevant to the review questions (nursing problems and outcomes), and reported adverse events. Any disagreements that arose between the reviewers were resolved through discussion with a third reviewer.

### Data Analysis and Presentation

To quantify the study results, parts of the extracted data were coded. A list of cancers was developed incorporating common and prevalent cancers retrieved from the National Cancer Institute [30] and the World Health Organization [31]. Digital interventions were categorized by type according to the initial categorization of digital interventions by Deloitte [4] and the categorization by Aapro et al [3], which supplemented the list developed by Deloitte [4]. The types of digital health interventions were categorized as *telemonitoring, telemedicine, wearables, web based, mobile apps, health analytics,* and *digitized health systems*. With regard to digital health interventions comprising multiple types—for example, *telemonitoring+telemedicine, wearable+web based,* and *web based+mobile*—each combination was considered a category. The National Institute for Health and Care Excellence (NICE) evidence standards framework for digital health technologies was used [32] to sort digital interventions into 3 tiers and 10 functional categories: tier A (*system services*), tier B (*inform, health diaries,* and *communicate*), and tier C (*preventative behavior change, self-manage, treat, active monitoring, calculate,* and *diagnose*). If a digital health intervention comprised multiple functions, then the function was classified as *multiple*.

Outcomes of digital health interventions were categorized as having positive, no, negative, mixed, or equivalent effects based on the purpose of the study. If significant outcomes were identified that corresponded with the hypothesis, they were categorized as having positive effects*.* Insignificant outcomes were categorized as having no effect. Significant opposite outcomes were considered negative outcomes. If the outcome was both positive and negative, it was considered as having a mixed effect. If the study aimed to demonstrate equivalent effectiveness, it was considered as having an equivalent effect. When the outcome was measured with multiple subscales and only partial outcomes were significant, then the study was categorized as having a positive effect. This also applied to longitudinal study outcomes where the same outcome was measured multiple times and a positive outcome identified at some of the time points. When the study had multiple comparisons, the outcomes of each comparison arm were identified.

With regard to summarizing the outcomes of the digital health interventions, if there were multiple digital health intervention studies targeting the same nursing problem, the outcomes were summarized and the effects compared. If the number of studies showing a positive effect outnumbered those with no effect or negative outcomes, the intervention was considered to suggest a positive outcome. If the same number of studies existed with both positive and no effect or negative outcomes, the intervention was considered to have inconsistent outcomes. If there were more studies that demonstrated no effect or negative outcomes, the intervention was considered to suggest no effect or negative outcome*.* A narrative and quantitative summary was accompanied by the tabulated and charted results.

## Results

### General Characteristics

**Figure 1:**
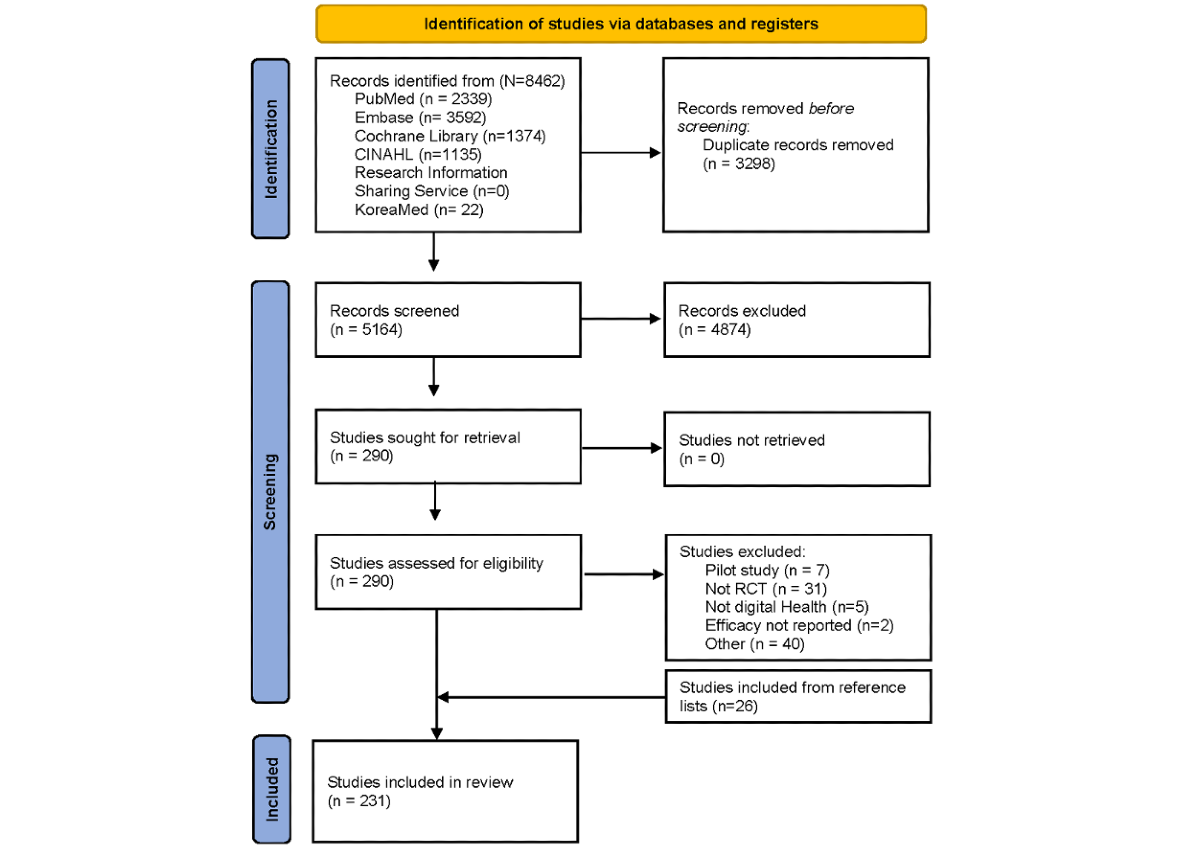
PRISMA-ScR (Preferred Reporting Items for Systematic Reviews and Meta-Analyses extension for Scoping Reviews) flow diagram. RCT: randomized controlled trial.

Of the 5164 articles screened, 231 (4.47%) [6,33-262] were selected and reviewed. The results of the search and the study inclusion process are presented in a PRISMA-ScR (Preferred Reporting Items for Systematic Reviews and Meta-Analyses extension for Scoping Reviews) flow diagram [263] (Figure 1).

Although digital health RCTs for patients with cancer continued to be reported, only a handful of studies were reported until 2012. Of the 231 included RCTs, 19 (8.2%) were published in 2013, whereas 50 (21.6%) were published in 2020. The United States was the country with the largest number of studies (119/231, 51.5%), followed by the Netherlands (26/231, 11.3%) and Australia (18/231, 7.8%).

Two-thirds of the studies were full-size RCTs and evaluated the effectiveness of digital health interventions (163/231, 70.6%), whereas the rest were pilot studies (68/231, 29.4%). Most of the studies were designed as 2-arm RCTs comparing the digital health interventions and control groups (206/231, 89.2%), but there were also 3-arm (22/231, 9.5%) and 4-arm (3/231, 1.3%) studies. Approximately half of the control groups received usual care or standard care (109/231, 47.2%). Waitlist control groups received the intervention after data collection was completed (47/231, 20.3%).

### Participant Characteristics

The number of participants included in the study (both pilot and full-size RCTs) ranged from 8 to 614 for the intervention group and 9 to 621 for the control group. In terms of age, digital interventions were mostly applied to adult patients with cancer (97/231, 42%) or both adult and geriatric patients with cancer (119/231, 51.5%), whereas studies targeting specifically older adults with cancer were scarce (6/231, 2.6%). More than half of the studies included adult patients with cancer of both sexes (124/231, 53.7%), whereas 38.5% (89/231) of the studies included only female patients with cancer. More than half of the studies were conducted with subjects with a single cancer (129/231, 55.8%), and among them, patients with breast cancer were the most frequently studied (79/129, 61.2%), followed by patients with prostate cancer (19/129, 14.7%) and patients with lung cancer (9/129, 7%). Patients with early-stage cancer were targeted in 38.5% (89/231) of the studies, and 32% (74/231) of the studies included patients with cancer of any stage, whereas 9.5% (22/231) of the studies targeted only patients with advanced or metastatic cancer. Studies about cancer survivors accounted for the highest percentage (97/231, 42%), followed by interventions provided along with cancer treatment (70/231, 30.3%).

### Types and Functions of Digital Health Interventions

**Figure 2:**
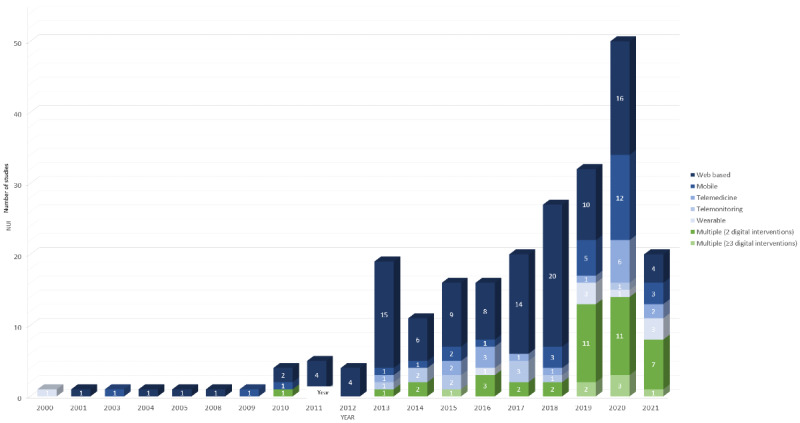
Types of digital health interventions (N=231).

Among the 231 studies, 196 (84.8%) included only digital interventions, whereas the rest (n=35, 15.2%) included digital interventions combined with nondigital interventions such as interventions provided in person (14/35, 40%), by telephone (10/35, 29%), by telephone+in person (10/35, 29%), and by CD-ROM multimedia program (1/35, 3%). The analysis based on digital health interventions showed that *web-based* digital health technology was the most frequently used type of digital intervention (116/231, 50.2%), followed by *mobile app* (31/231, 13.4%), *telemedicine* (17/231, 7.4%), *telemonitoring* (11/231, 4.8%), and *wearable* (9/231, 3.9%). Digital interventions comprising multiple modalities accounted for 20.3% (47/231) of the interventions, in which *web based+mobile app* was the most frequently used modality (23/47, 49%), followed by *wearable+mobile app* (9/47, 19%), *wearable+web based+mobile app* (5/47, 11%), *telemonitoring+telemedicine* (3/47, 6%), *wearable+web based* (2/47, 4%), *telemedicine+web based* (2/47, 4%), *telemedicine+mobile app* (1/47, 2%), *telemedicine+web based+mobile app* (1/47, 2%), and *telemonitoring+wearable+web based+mobile app* (1/47, 2%; Figure 2).

Digital health interventions consisting of *multiple* functional components (69/231, 29.9%) were the most frequently used interventions, followed by interventions for *self-manage* (67/231, 29%). Among the studies that applied digital interventions with *multiple* components, *communicate+self-manage* (18/69, 26%) was the frequently identified combination.

**Figure 3:**
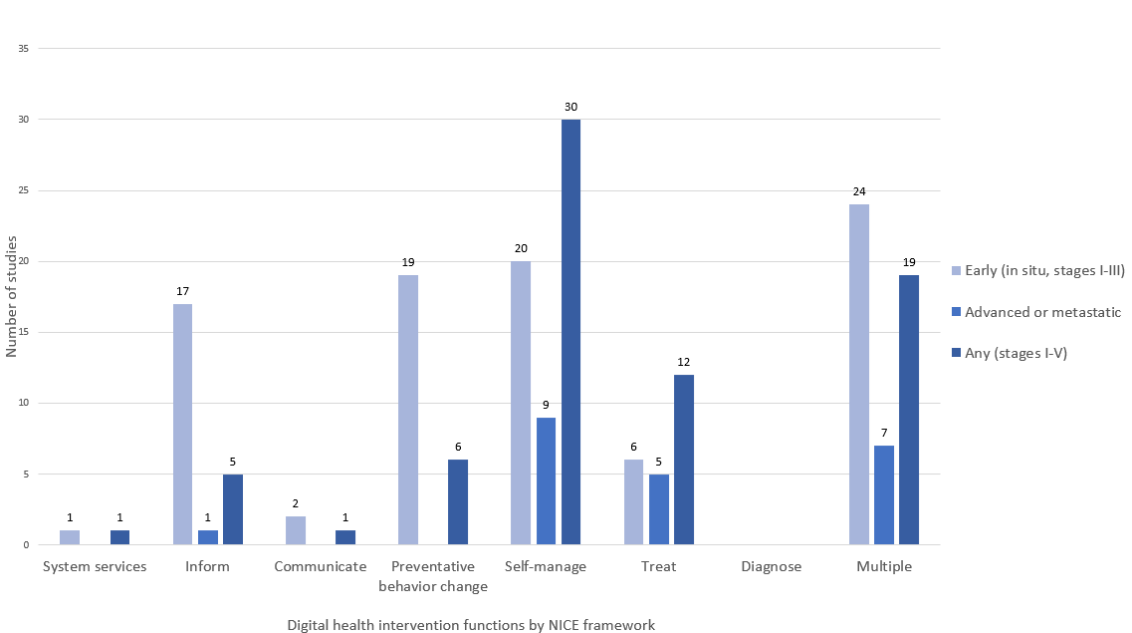
Digital health intervention functions by stage (n=185). NICE: National Institute for Health and Care Excellence.

**Figure 4:**
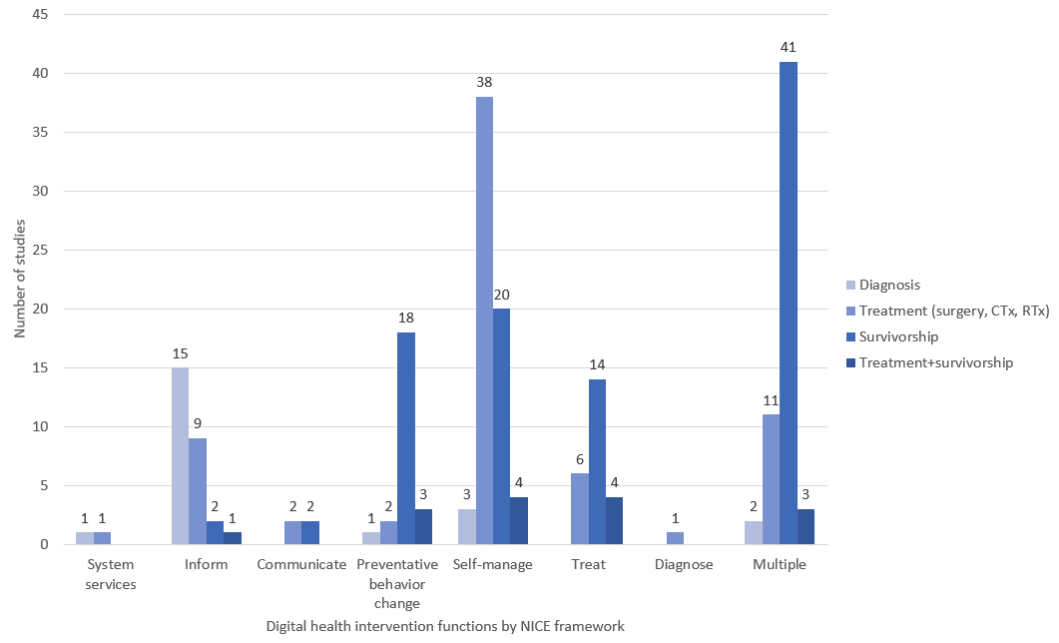
Digital health intervention functions by phase (n=204). CTx: chemotherapy; NICE: National Institute for Health and Care Excellence; RTx: radiation therapy.

*Self-manage* was the most prevalent single function of digital health function regardless of patients’ cancer stages (185/231, 80.1%; Figure 3). With regard to patients’ cancer journeys, *inform* was usually applied at the diagnosis phase. *Self-manage* interventions during the treatment phase accounted for the largest proportion (38/70, 54%) of the provided interventions. Interventions with *multiple* components mostly targeted the survivorship phase (41/97, 42%), which comes after the treatment phase (11/97, 11%; Figure 4).

### Frequency, Duration, and Time Spent Administering Interventions

Most of the digital interventions were applied at home (187/231, 81%). There were several studies that did not report the frequency of application of digital interventions (129/231, 55.8%). Among those that reported the frequency of application (102/231, 44.2%), weekly application accounted for 34.3% (35/102) of the interventions, whereas daily application accounted for 8.8% (9/102). The duration of application ranged from 2 weeks to 144 weeks (152/231, 54.1%); the 12-week application was the most frequent (43/156, 27.6%), whereas 8-week and 24-week periods were applied in 14.7% (23/156) each of the interventions. In most (181/231, 78.4%) of the cases, the application time per single intervention was not reported. The average application time among the studies that reported information about application time per single intervention was approximately 60 (mean 63.92, SD 49.71; range 5-240) minutes (50/231, 21.6%).

### Targeted Nursing Problems and Outcomes

Data from full-size RCTs were used to summarize targeted nursing problems and outcomes of digital health interventions.

To better understand the outcomes of the digital health interventions, outcomes identified from >2 studies with the same target, type, and function were summarized (Table 1). *Wearable* with *multiple*-*function* interventions for physical activity all demonstrated positive outcomes. *Web-based* interventions to *inform* patients to intervene in anxiety; *preventative behavior change* for physical activity; *self-manage* symptoms; *treat* for cognitive function, depression, fear of cancer recurrence, and sleep; and those with *multiple* functional components for symptoms all demonstrated positive outcomes. *Web-based* interventions combined with *mobile multiple-function* interventions all demonstrated positive outcomes for pain. *Telemonitoring* interventions combined with *telemedicine* and *treat* demonstrated positive outcomes for patients with depression as well as pain.

**Table 1:** Types, functions, and effects of digital health interventions for nursing problems.

Problems, digital health type, and digital health function				Positive effect		No effect		Negative effect		Mixed effect^a^		Equivalent effect
**Adherence: medication**												
	**Mobile**											
		Self-manage	[133,210]		[108,116]		N/A^b^		N/A		N/A	
**Anxiety**												
	**Web**											
		Inform	[138,164,195]		N/A		N/A		N/A		N/A	
		Treat	[163]		[82]		N/A		N/A		[79]	
		Multiple^c^	[57]^d^ [234]^d,e^		[170,225,227]		N/A		N/A		N/A	
	**Web+mobile**											
		Multiple	[117] [205]^e^ [231]^f^		[58]^g^ [173]		N/A		N/A		N/A	
**BMI**												
	**Web**											
		Multiple	[97]^e^		[234]^d,e^		N/A		N/A		N/A	
**Breast cancer concerns**												
	**Web**											
		Multiple	[114]^d,e,h^		[44]^d^ [114]^d,e,h^		N/A		N/A		N/A	
**Cancer-specific distress**												
	**Web**											
		Self-manage	[218]		[46,47] [208]^d^^,^^e^		N/A		N/A		N/A	
**Cognitive function**												
	**Web**											
		Treat	[82,158]		N/A		N/A		N/A		N/A	
**Communication with provider**												
	**Web**											
		Self-manage	[49]		[216]		N/A		N/A		N/A	
**Competence: health care**												
	**Web**											
		Multiple	[44]^d^ [115]^d^^,^^e^		[44]^d^ [114]^d,e,h^ [115]^d^^,^^e^		N/A		N/A		N/A	
**Competence: information**												
	**Web**											
		Multiple	[44]^d^ [114]^d,e,h^ [134]^d,^^e^		[44]^d^ [114]^d,e,h^ [115]^d^^,^^e^		N/A		N/A		N/A	
**Coping**												
	**Web**											
		Self-manage	[180,209]		[46,47]		N/A		N/A		N/A	
		Multiple	[44]^d^ [115]^d^^,^^e^		[44]^d^ [114]^d,e,h^ [115]^d^^,^^e^		N/A		N/A		N/A	
**Cost**												
	**Telemedicine**											
		Treat	[148]		[152]		N/A		N/A		N/A	
	**Web**											
		Self-manage	N/A		[34]		N/A		N/A		[219]	
**Decision-making**												
	**Web**											
		Inform	[50] [78]^i^ [243,247,262]		[73,84,157,175]		N/A		N/A		N/A	
**Depression**												
	**Web**											
		Self-manage	[86,142,196,203]		[208,234]^d,e^		N/A		N/A		N/A	
		Treat	[82,163] [237]^g^		N/A		N/A		N/A		[79]	
		Multiple	[57]^d^ [227]		[54,170,183] [204]^g^ [208]^d^^,^^e^ [225,228] [234]^d^^,^^e^ [240]		N/A		N/A		N/A	
	**Web+mobile**											
		Multiple	[117]		[58]^g^ [173] [205]^e^		N/A		N/A		N/A	
	**Telemonitoring+telemedicine**											
		Treat	[140,244]		N/A		N/A		N/A		N/A	
**Diet**												
	**Web**											
		Multiple	N/A		[127,240]		N/A		N/A		N/A	
**Distress**												
	**Web**											
		Communicate	N/A		N/A		[119,144]		N/A		N/A	
		Self-manage	[142] [189]^f^ [212,218,233]		[35,46,47,193]		N/A		N/A		N/A	
		Treat	[33]^g^ [72,163] [217]^g^		[41]^d,e^ [158]		N/A		N/A		N/A	
		Multiple	[76,229]		[59]^d^ [75] [97]^e^ [102]		N/A		N/A		N/A	
	**Mobile**											
		Multiple	[68]		[155]		N/A		N/A		N/A	
**Emotional processing**												
	**Web**											
		Multiple	[44]^d^ [114]^d,e,h^		[44]^d^ [114]^d,e,h^		N/A		N/A		N/A	
**Fatigue**												
	**Web**											
		Self-manage	[196,218,233]		[208]^d,e^ [234]^d^		N/A		N/A		N/A	
		Treat	[33,217]^g^ [235] [237]^g^		[82,158]		N/A		N/A		N/A	
		Multiple	[59]^d^ [170,227]		[54] [204]^g^ [208]^d^^,^^e^ [228] [234]^d,e^ [240]		N/A		N/A		N/A	
	**Web+mobile**											
		Multiple	[117]		[238]		N/A		N/A		N/A	
**Fear of cancer recurrence or progression**												
	**Web**											
		Self-manage	[218]		[221]		N/A		N/A		N/A	
		Treat	[72,163] [217]^g^		N/A		N/A		N/A		[79]	
**Follow-up**												
	**Telemedicine**											
		Treat	[40]		[152]		N/A		N/A		N/A	
**Function**												
	**Web**											
		Self-manage	N/A		[86,180] [208]^d^^,^^e^		N/A		N/A		N/A	
		Multiple	[39]		[170] [208]^d^^,^^e^		N/A		N/A		N/A	
**Functional well-being**												
	**Web**											
		Self-manage	N/A		[62,209]		N/A		N/A		N/A	
		Multiple	[114]^d,e,h^ [115]^d,e^		[44]^d^ [114]^d,e,h^ [115]^d,e^		N/A		N/A		N/A	
**HRQoL^j^/QoL^k^**												
	**Telemonitoring**											
		Self-manage	[90,174]		[136,232]		N/A		N/A		N/A	
	**Telemedicine**											
		Multiple	[207]		[94]^e^		N/A		N/A		N/A	
	**Web**											
		Inform	[138]^h^ [195]		[175]		N/A		N/A		N/A	
		Self-manage	[34,46] [189]^f^ [212,220,233]		[35,47,218] [261]^h^		N/A		N/A		N/A	
		Treat	[33]^g^ [71,72,163] [217]^g^		[41]^d^^,^^e^ [82]		N/A		N/A		[79]	
		Multiple	[97]^e^ [102,168] [204]^g^ [225,227]		[54] [115]^d,e^ [183] [197]^e^ [228,249,251]		N/A		N/A		N/A	
	**Mobile**											
		Self-manage	[118]		[108,260]		N/A		N/A		N/A	
		Treat	[199]		[107]		N/A		N/A		N/A	
		Multiple	[68]		[155]		N/A		N/A		N/A	
	**Web+mobile**											
		Multiple	[58]^g^ [117,120] [231]^f^ [238]		[173,214]		N/A		N/A		N/A	
	**Telemonitoring+telemedicine**											
		Treat	[65]^d^ [140,244]		[65]^d^		N/A		N/A		N/A	
**Intrusive thoughts**												
	**Web**											
		Self-manage	N/A		[142,203]		N/A		N/A		N/A	
**Knowledge**												
	**Web**											
		Inform	[73,105,157,175,194,211,262]		[84,164]		N/A		[186]		N/A	
		Multiple	[251]		[102]		N/A		N/A		N/A	
**Mood**												
	**Web**											
		Self-manage	[203]		[62]		N/A		N/A		N/A	
**Pain**												
	**Web**											
		Self-manage	N/A		[196,233]		N/A		N/A		N/A	
		Multiple	N/A		[64] [204]^g^		N/A		N/A		N/A	
	**Web+mobile**											
		Multiple	[173,238]		N/A		N/A		N/A		N/A	
	**Telemonitoring+telemedicine**											
		Treat	[65]^d^ [140]		N/A		N/A		N/A		N/A	
**Physical activity**												
	**Wearable**											
		Multiple	[113]^e^ [150]^g^		N/A		N/A		N/A		N/A	
	**Web**											
		Preventative behavior change	[103,250]		N/A		N/A		N/A		N/A	
		Self-manage	N/A		[233] [234]^d,^^e^		N/A		N/A		N/A	
		Multiple	[97]^e^ [128,240]		[89]^d^ [127] [234]^d,^^e^		N/A		N/A		N/A	
	**Web+mobile**											
		Multiple	N/A		[117,214]		N/A		N/A		N/A	
**Personal control**												
	**Web**											
		Self-manage	N/A		[218,220]		N/A		N/A		N/A	
**Recall of cancer-related information**												
	**Web**											
		Inform	[55]^d^		[55,56]^d^		N/A		N/A		N/A	
**Self-efficacy**												
	**Web**											
		Inform	N/A		[101,164]		N/A		N/A		N/A	
		Self-manage	[34,62,218]		[196,220]		[209]		N/A		N/A	
		Multiple	[251]		[57]^d^ [64,183] [197]^e^		N/A		N/A		N/A	
	**Web+mobile**											
		Multiple	[231]^f^		[63]		N/A		N/A		N/A	
**Sleep**												
	**Web**											
		Treat	[41]^d,e^ [235] [237]^g^		N/A		N/A		N/A		N/A	
	**Web+mobile**											
		Multiple	[117]		[173,238]		N/A		N/A		N/A	
**Social support**												
	**Web**											
		Multiple	[114]^d^^,^^e^^,^^h^ [249]		[44]^d^ [114]^d,e,h^ [115]^d^^,^^e^ [183] [234]^d,e^		N/A		N/A		N/A	
**Symptom**												
	**Telemonitoring**											
		Self-manage	[70,90,136,162,174]		[161,232]		N/A		N/A		N/A	
	**Web**											
		Self-manage	[34,51,62,180,220,257]		N/A		N/A		N/A		N/A	
		Multiple	[57]^d^ [110,183]		N/A		N/A		N/A		N/A	
	**Mobile**											
		Self-manage	[179,184]		[108,260]		N/A		[129]		N/A	

^a^Mixed effect: both positive and negative effects were identified.

^b^N/A: not applicable.

^c^Multiple: multiple digital health functions were combined.

^d^Multiple-arm study and different intervention groups.

^e^Additional intervention was provided by telephone (provided only in some arms in multiple-arm study).

^f^Additional intervention was provided in person.

^g^Additional intervention was provided in person and by telephone.

^h^Multiple-arm study and different control groups.

^i^Additional intervention was provided by CD-ROM.

^j^HRQoL: health-related quality of life.

^k^QoL: quality of life.

More studies with positive outcomes were identified for *web-based* interventions to *inform* decision-making, HRQoL or quality of life (QoL), and knowledge; to *self-manage* depression, distress, fatigue, and HRQoL or QoL; to *treat* distress, fatigue, and for HRQoL or QoL; and *web+mobile multiple-function* interventions for anxiety and HRQoL or QoL. More studies with positive outcomes were also identified for *telemonitoring* to *self-manage* symptoms and *telemonitoring* combined with *telemedicine* and *treat* for HRQoL or QoL.

Studies with no effect or negative outcomes outnumbered *web-based* interventions to *inform* for recall of cancer-related information; *self-manage* interventions for cancer-specific distress and cost; and the following *multiple*-*function* interventions for anxiety, breast cancer concerns, and competence: health care, coping, depression, distress, fatigue, function, functional well-being, HRQoL or QoL, self-efficacy, and social support. There were also a greater number of studies with no effect or negative outcomes with regard to *mobile self-manage* interventions for HRQoL or QoL and *web-based* interventions combined with *mobile multiple-function* interventions for depression and sleep.

No effects were reported for *web-based* interventions to *inform* targeting self-efficacy or *self-manage* interventions for function, functional well-being, intrusive thoughts, pain, physical activity, and personal control. *Web-based multiple*-*function* interventions targeting diet and pain demonstrated no effects. *Web-based* interventions combined with *mobile* interventions with *multiple-function* interventions for physical activity also demonstrated no effects.

Notably, the effectiveness of digital health interventions targeting anxiety, depression, distress, fatigue, HRQoL or QoL, pain, physical activity, and sleep depended on the type and function of the interventions.

Two *web-based* interventions with *communicate* functions for distress only demonstrated negative outcomes. Inconsistent outcomes from equivalent numbers of studies with positive effects and no effect or negative effects were identified from the rest of the studies listed (Table 2).

Adverse events were reported in some of the studies [33,72,77,79,117,173,218,251]. There was increased distress because the information content reminded patients of their cancer, because patients felt pressured to engage in healthy behavior, and because patients felt frustrated by options that did not match their situation [117]. In addition, increased symptoms [77,79] and unintended weight loss while trying a healthy diet [117] were reported as adverse events. Some of the adverse events that were unrelated to the digital intervention [72] occurred in 2 participants in the usual-care group who were concerned about cancer recurrence [33] and in the control group participants who played the control game [251] or were similar for both the intervention and control groups [173]. A single serious adverse event occurred, in which 1 participant was admitted to the psychiatric clinic; however, the study did not report whether this event occurred in the intervention group or control group [218].

**Table 2:** Summary of digital health intervention outcomes.

Types of digital health interventions	Studies with positive outcomes	Studies with positive outcomes outnumbered studies with no effect or negative outcomes	Effectiveness of digital health interventions was subject to their type and function	Studies with no effect or negative outcomes outnumbered studies with positive outcomes	Studies with no effect	Studies with negative outcomes
Telemonitoring	N/A^a^	Self-manage symptom	Anxiety; depression; distress; fatigue; HRQoL^b^ or QoL^c^; pain; physical activity; sleep	N/A	N/A	N/A
Telemedicine	N/A	N/A	Anxiety; depression; distress; fatigue; HRQoL or QoL; pain; physical activity; sleep	N/A	N/A	N/A
Wearable	Multiple^d^ functions for physical activity	N/A	Anxiety; depression; distress; fatigue; HRQoL or QoL; pain; physical activity; sleep	N/A	N/A	N/A
Web	Inform for anxiety; preventative behavior change for physical activity; self-manage symptoms; treat for cognitive function, depression, fear of cancer recurrence, and sleep; multiple functions for symptom	Inform for decision-making, HRQoL or QoL, and knowledge; self-manage depression, distress, fatigue, and HRQoL or QoL; treat for distress, fatigue, and HRQoL or QoL	Anxiety; depression; distress; fatigue; HRQoL or QoL; pain; physical activity; sleep	Inform for recall of cancer-related information; self-manage intervention for cancer-specific distress and cost; multiple functions for anxiety, breast cancer concerns, and competence (including health care, coping, depression, distress, fatigue, function, functional well-being, HRQoL or QoL, self-efficacy, and social support)	Inform for self-efficacy; self-manage for function, functional well-being, intrusive thoughts, pain, physical activity, and personal control; multiple functions for diet and pain	Communicate for distress
Mobile	N/A	N/A	Anxiety; depression; distress; fatigue; HRQoL or QoL; pain; physical activity; sleep	Self-manage for HRQoL or QoL	N/A	N/A
Web+mobile	Multiple functions for pain	Multiple functions for anxiety and HRQoL or QoL	Anxiety; depression; distress; fatigue; HRQoL or QoL; pain; physical activity; sleep	Multiple functions for depression and sleep	Multiple functions for physical activity	N/A
Telemonitoring+telemedicine	Treat depression and pain	Treat for HRQoL or QoL	Anxiety; depression; distress; fatigue; HRQoL or QoL; pain; physical activity; sleep	N/A	N/A	N/A

^a^N/A: not applicable.

^b^HRQoL: health-related quality of life.

^c^QoL: quality of life.

^d^Multiple: multiple digital health functions were combined.

## Discussion

### Principal Findings

This scoping review provides a comprehensive summary of digital health interventions for adult patients with cancer or cancer survivors. A total of 231 studies that used digital health interventions were included in the review. By summarizing the broad literature, the major results showed that (1) *web-based* digital health technologies are the most frequently used intervention modality (116/231, 50.2%), (2) digital health interventions with *multiple* functions are the most studied (69/231, 29.9%), and (3) systematic reviews and studies regarding the clinical application of digital health interventions for patients with cancer are worthy of consideration. The effectiveness of digital health interventions targeting anxiety, depression, distress, fatigue, HRQoL or QoL, pain, physical activity, and sleep is subject to the type and function of the interventions, and this needs to be investigated further to select appropriate types and functions of digital health approaches depending on the targets. Digital health interventions that demonstrate inconsistent outcomes, as well as studies with no effect or negative outcomes that outnumber studies with positive outcomes, are worthy of further investigation, considering the limited number of studies conducted thus far. Digital health interventions that only demonstrated no effect or negative outcomes would have low priority for further investigation.

The number of RCTs using digital health interventions for patients with cancer has increased since 2013, and 50 RCT studies were published in 2020. The number of RCTs using digital health interventions conducted in the United States has been overwhelmingly high in the last 20 years. Since eHealth was first defined [29], it has gradually expanded owing to the change in the medical paradigm, the impact of the Fourth Industrial Revolution, and the popularization of smartphones.

In this review, digital interventions were mostly applied to adult patients (97/231, 42%) or both adult and older adult patients with cancer (119/231, 51.5%). However, digital health intervention studies targeting only geriatric patients with cancer were the rarest (6/231, 2.6%). Cancer is considered a disease of aging. Most of the new cancer diagnoses occur in older adults (aged >65 years) [264], and the incidence of new cancers is expected to double by 2035 among older adults [265]. For these reasons, providing high-quality care for older adults with cancer can be a high-priority item on the clinical agenda. In addition, cancer mortality has decreased, and the 5-year relative survival rate for all cancers has gradually increased to nearly 70% [266]. Considering the number of aging patients with cancer and the increasing number of cancer survivors, it is necessary to suggest directions for how to apply digital health interventions for older adult patients with cancer. As Kemp et al [267] suggested, the variability of digital health literacy in patients with cancer, including age and life circumstances, should be addressed to implement digital health interventions effectively and safely.

More than half (124/231, 53.7%) of the studies in this scoping review included both female and male patients with cancer. However, 38.5% (89/231) of the studies included only female patients with cancer, which is related to the fact that, of the 129 studies for single cancer, 79 (61.2%) were conducted on patients with breast cancer. A previous scoping review of 151 publicly available apps for cancer survivors found that a majority of the apps targeted all cancer types, followed by apps targeting breast cancer [268].

When applying digital health interventions, researchers should prioritize cancer types for which there is sufficient evidence indicating that these interventions are safe and effective. However, more studies are still needed to test their applicability to specific cancer types and broaden the range of cancer types to which they can be applied. Almost three-fourths (163/231, 70.6%) of the studies in this review involved patients with early-stage cancer or patients with cancer of any stage, but only 9.5% (22/231) of the studies targeted only patients with advanced or metastatic cancer. Digital health interventions for cancer survivors accounted for the largest proportion (97/231, 42%), followed by digital health interventions provided along with cancer treatment (70/231, 30.3%). Further studies are particularly required on the direction of digital health interventions for patients with advanced or metastatic cancer because studies of patients with advanced or metastatic cancer in the later stages of life or of caregivers of palliative care reported more unmet needs than studies that included cancer survivors [269].

Most (187/231, 81%) of the digital interventions were applied at home. The clinical context for cancer treatment has shifted to outpatient or home settings in recent years, and digital health technology can be one of the solutions to support patients with cancer in their care continuum [270]. It is expected that the number of digital health interventions applied at home by patients with cancer and their caregivers will increase in the future. As this scoping review focused on digital health interventions for patients with cancer, digital health studies about *system services*, which did not have intervention components, might have been rare.

Digital health interventions using *web-based* methods have been the most frequently evaluated during the past 20 years. Considering their universality and accessibility, it is natural that *web-based* digital health interventions have been the most studied. Before developing digital health interventions for patients with cancer, the key factors affecting their use should be considered from the patients’ perspective. Aapro et al [3] outlined 7 factors affecting the uptake of digital tools from the patients’ perspective: ease of use, reassurance, high usability and usefulness, improved communication with health care professionals, correct generation of system alerts and fast response to alerts, patient empowerment, and the convenience of real-time reporting of symptoms. Moreover, with the popularization of smartphones, the accessibility of apps has increased, and patients with cancer and their caregivers can easily find some apps related to cancer. Adam et al [268] found that most of the publicly available apps for cancer survivors were developed by commercial or private organizations, and judging quality, effectiveness, clinical utility and data protection issues could be challenging for cancer survivors and health care providers. Researchers need to consider issues specific to digital health in developing and evaluating interventions for patients with cancer.

Among the NICE functional categories, interventions comprising *multiple* functions were the most frequently studied (69/231, 29.9%), followed by interventions comprising *self-manage* functions (67/231, 29%). Digital health interventions with the *self-manage* function provided options for users to record and send data to a health care professional [32]. Digital health interventions for symptom tracking combined with feedback from health care professionals are good candidates for clinical translation. The use of patient-reported outcome measures can improve health professional–patient communication, symptom management, supportive care, and patient satisfaction [271]. *Health diaries* or *active monitoring* were not used as stand-alone features of the interventions in this study, although they were used as part of *multiple* functions. *Calculate* refers to “tools that perform clinical calculations that are likely to affect clinical care decisions,” and digital health interventions classified into the *calculate* category are generally used by clinicians or professionals [32]. As this review concerned digital health interventions for patients with cancer, no studies were identified for the *calculate* function.

Effectiveness could vary in relation to patient population and difference in technology [25], and this review showed heterogeneous outcomes by type and function of the interventions. *Web-based self-manage* interventions for communicating with providers, *web-based self-manage* and *multiple*-*function* interventions targeting symptoms, and *web-based* interventions to *treat* cognitive function and fear of cancer recurrence showed consistent positive outcomes in this scoping review. Although different types and functions of digital health interventions could result in different outcomes, positive outcomes were also demonstrated by *web-based* interventions to *inform* anxiety, *web-based* as well as *telemonitoring* interventions combined with *telemedicine* interventions to *treat* depression, *web-based* interventions combined with *mobile* interventions with *multiple* functions and *telemonitoring* combined with *telemedicine* interventions to *treat* pain, *wearable* with *multiple* functions and *web-based* interventions for *preventative behavior change* for physical activity, and *web-based* interventions to *treat* sleep. A systematic review evaluating digital health interventions providing supportive care for patients with cancer supported a positive effect on symptoms such as fatigue, pain, and depression [25]. Moreover, more studies with positive outcomes were identified in this scoping review for *web-based* interventions to *inform*, *self-manage*, and *treat* for HRQoL or QoL; *web+mobile multiple*-*function* interventions for HRQoL or QoL; and *telemonitoring* combined with *telemedicine* to *treat* for HRQoL or QoL. Positive outcomes were identified in some previous reviews that were narrower than this scoping review and focused on specific cancer types such as prostate cancer or, predominantly, breast cancer, indicating the effect of the intervention on HRQoL [14,25]. The results of this review, which has a broader scope in terms of population, showed some positive effects and some null effects. Further systematic study is needed to clarify whether the difference in outcomes arises from patient characteristics or from the intervention. Many previous reviews that evaluated the effectiveness of digital interventions for patients with cancer provided inconsistent and inconclusive results and suggested that well-planned further studies should be conducted; for example, Escriva Boulley et al [26] systematically reviewed the engagement with digital health interventions of patients with cancer and the psychosocial effects of the interventions in 29 articles (24 studies), and the efficacy of digital health interventions in changing psychosocial outcomes was inconsistent. The findings of the study by Roberts et al [23] indicated that digital behavior change interventions for cancer survivors might improve physical activity and BMI, but evidence for diet was mixed. The mixed outcomes for symptom management using the mobile app intervention included the positive effect of lower levels of fatigue and an increase in the number of reports of hand-foot syndrome in the intervention arm. Facilitation of symptom assessment by the mobile app intervention might have contributed to the mixed outcomes [129]. Another set of mixed outcomes concerned knowledge, where the control group reported increased perception of received knowledge, whereas the intervention group reported a decrease in perception of received knowledge [186]. Higher perception of received knowledge among control group participants was interpreted as deriving from patients’ satisfaction with the education they received from hospital staff in person. When knowledge was evaluated in terms of the knowledge level rather than the perception of received knowledge, participants in the internet-based patient education program demonstrated a higher level of knowledge. The effect of a personal touch and the effectiveness of digital interventions in increasing knowledge might have resulted in these mixed outcomes. Overall, digital health interventions targeting cognitive function, mindfulness, and strength all demonstrated positive outcomes; however, we did not include mindfulness [72,259] and strength [39,80,96] in Tables 1 and 2 because only a single study existed for each digital health intervention type and function. Future development of digital health interventions and RCTs would provide enriched resources to understand the effectiveness of digital health interventions for patients with cancer.

A negative effect of *web-based communicate* interventions on distress [119,144] and of *web-based self-manage* interventions on self-efficacy [209] as well as a few adverse events that occurred while conducting digital health studies were identified. The influence of digital health modalities such as playing games on dizziness needs to be considered, although the reported adverse events occurred in a control group participant. An increase in distress and symptoms and unintended weight loss could be related to intervention content rather than the digital delivery of the intervention, and this needs to be taken into consideration in developing the contents of future digital health interventions for patients with cancer.

The use of digital health interventions would be inevitable when considering the advances in digital technology and the needs of patients with cancer, caregivers, and health care professionals. Health care providers should help to develop appropriate digital health interventions for patients with cancer because engaging patients with cancer in their care can make for better health outcomes, and this is also expected to decrease health care providers’ burden [272]. To develop digital health interventions that qualify as both efficacious and safe for patients with cancer, the collaboration of a multidisciplinary team will be necessary. The strengths of this study include (1) providing a comprehensive overview of digital health interventions for adult patients with cancer or cancer survivors from the time when the definition of eHealth was first introduced to the present day and (2) categorizing digital interventions by type and function and summarizing outcomes, which enables the identification of digital health interventions requiring further investigations and systematic reviews.

### Limitations

We applied the highest standard with regard to research design (RCTs) in selecting digital health interventions considering patient characteristics (patients with cancer). According to the NICE evidence standards framework, only tier C digital health interventions for treatment (specifically for *treat*, *active monitoring*, *calculate*, or *diagnose* functions) require a high-quality RCT as the best practice standard [32]. This study was limited because it included only RCTs. The most frequently identified functions of digital health interventions in this scoping review—*multiple* and *self-manage—*could be evaluated using a study design with a broader scope, including a high-quality quasi-experimental study design with comparison groups; thus, in this scoping review, areas that considered gaps in research could have been overestimated. With regard to outcomes of digital health interventions, outcomes identified from >2 studies of the same target, type, and function were summarized. Of note, there were single well-designed full-size RCTs that demonstrated the effectiveness of digital health interventions of specific types and functions targeting nursing problems, which were not included in Table 1.

### Conclusions

This scoping review investigated the types and functions of digital health interventions developed and evaluated for patients with cancer that targeted various nursing problems and summarized their outcomes. Systematic reviews based on this scoping review—for example, a systematic review on the efficacy of interventions in relation to patient characteristics—should be the next step to accumulate evidence regarding the clinical application of digital health interventions for patients with cancer. The relationship between the effectiveness of digital health interventions and their types and functions needs to be investigated further to guide the selection of appropriate types and functions of digital health approaches depending on the targets. Digital health interventions that demonstrate equivalent outcomes, as well as those for which no effect or negative outcomes outnumber positive outcomes in the literature, are worthy of further investigation, considering the limited number of studies conducted. The identified gaps in digital health resources for cancer care need to be reflected in future digital health research.

## Appendix

Multimedia Appendix 1 Search strategy for PubMed.

## Abbreviations

HRQoL: health-related quality of life
NICE: National Institute for Health and Care Excellence
PRISMA-ScR: Preferred Reporting Items for Systematic Reviews and Meta-Analyses extension for Scoping Reviews
QoL: quality of life
RCT: randomized controlled trial

## References

[1] Eysenbach G (2001). What is e-health?. J Med Internet Res.

[2] Ronquillo Y, Meyers A, Korvek S (2022). Digital health. StatPearls.

[3] Aapro M, Bossi P, Dasari A, Fallowfield L, Gascón P, Geller M, Jordan K, Kim J, Martin K, Porzig S (2020). Digital health for optimal supportive care in oncology: benefits, limits, and future perspectives. Support Care Cancer.

[4] (2015). Digital health in the UK: an industry study for the office of life sciences. Deloitte.

[5] (2019). WHO Guideline: Recommendations on Digital Interventions for Health System Strengthening.

[6] Basch E, Deal AM, Kris MG, Scher HI, Hudis CA, Sabbatini P, Rogak L, Bennett AV, Dueck AC, Atkinson TM, Chou JF, Dulko D, Sit L, Barz A, Novotny P, Fruscione M, Sloan JA, Schrag D (2016). Symptom monitoring with patient-reported outcomes during routine cancer treatment: a randomized controlled trial. J Clin Oncol.

[7] Archer S, Cheung NH, Williams I, Darzi A (2021). The impact of digital health interventions on the psychological outcomes of patients and families receiving paediatric palliative care: a systematic review and narrative synthesis. Palliat Med.

[8] Cheng L, Duan M, Mao X, Ge Y, Wang Y, Huang H (2021). The effect of digital health technologies on managing symptoms across pediatric cancer continuum: a systematic review. Int J Nurs Sci.

[9] Hong HC, Min A, Kim YM (2021). The effectiveness of digital self-management interventions on health outcomes among childhood cancer survivors: a systematic review and meta-analysis. J Adv Nurs.

[10] McCann L, McMillan KA, Pugh G (2019). Digital interventions to support adolescents and young adults with cancer: systematic review. JMIR Cancer.

[11] Ramsey WA, Heidelberg RE, Gilbert AM, Heneghan MB, Badawy SM, Alberts NM (2020). eHealth and mHealth interventions in pediatric cancer: a systematic review of interventions across the cancer continuum. Psychooncology.

[12] Viola A, Panigrahi G, Devine KA (2020). Digital interventions for adolescent and young adult cancer survivors. Curr Opin Support Palliat Care.

[13] Sotirova MB, McCaughan EM, Ramsey L, Flannagan C, Kerr DP, O'Connor SR, Blackburn NE, Wilson IM (2021). Acceptability of online exercise-based interventions after breast cancer surgery: systematic review and narrative synthesis. J Cancer Surviv.

[14] Forbes CC, Finlay A, McIntosh M, Siddiquee S, Short CE (2019). A systematic review of the feasibility, acceptability, and efficacy of online supportive care interventions targeting men with a history of prostate cancer. J Cancer Surviv.

[15] Rollin A, Ridout B, Campbell A (2018). Digital health in melanoma posttreatment care in rural and remote Australia: systematic review. J Med Internet Res.

[16] Shah AC, O'Dwyer LC, Badawy SM (2021). Telemedicine in malignant and nonmalignant hematology: systematic review of pediatric and adult studies. JMIR Mhealth Uhealth.

[17] Bradbury K, Steele M, Corbett T, Geraghty AWA, Krusche A, Heber E, Easton S, Cheetham-Blake T, Slodkowska-Barabasz J, Müller AM, Smith K, Wilde LJ, Payne L, Singh K, Bacon R, Burford T, Summers K, Turner L, Richardson A, Watson E, Foster C, Little P, Yardley L (2019). Developing a digital intervention for cancer survivors: an evidence-, theory- and person-based approach. NPJ Digit Med.

[18] Mikles SP, Griffin AC, Chung AE (2021). Health information technology to support cancer survivorship care planning: a systematic review. J Am Med Inform Assoc.

[19] Karamanidou C, Natsiavas P, Koumakis L, Marias K, Schera F, Schäfer M, Payne S, Maramis C (2020). Electronic patient-reported outcome-based interventions for palliative cancer care: a systematic and mapping review. JCO Clin Cancer Inform.

[20] Salvador Vergès À, Cusí Sánchez MV, Bossio Grigera P, Fàbrega Agulló C, Gomes da Costa F, Serra Trullas A, García Abejas A (2022). Determinants in stakeholder opinions about telemedicine in palliative care: a scoping review. Telemed J E Health.

[21] Gambalunga F, Iacorossi L, Notarnicola I, Serra V, Piredda M, De Marinis MG (2020). Mobile health in adherence to oral anticancer drugs: a scoping review. Comput Inform Nurs.

[22] Schaffer K, Panneerselvam N, Loh KP, Herrmann R, Kleckner IR, Dunne RF, Lin P, Heckler CE, Gerbino N, Bruckner LB, Storozynsky E, Ky B, Baran A, Mohile SG, Mustian KM, Fung C (2019). Systematic review of randomized controlled trials of exercise interventions using digital activity trackers in patients with cancer. J Natl Compr Canc Netw.

[23] Roberts AL, Fisher A, Smith L, Heinrich M, Potts HWW (2017). Digital health behaviour change interventions targeting physical activity and diet in cancer survivors: a systematic review and meta-analysis. J Cancer Surviv.

[24] Hong YA, Hossain MM, Chou WS (2020). Digital interventions to facilitate patient-provider communication in cancer care: a systematic review. Psychooncology.

[25] Marthick M, McGregor D, Alison J, Cheema B, Dhillon H, Shaw T (2021). Supportive care interventions for people with cancer assisted by digital technology: systematic review. J Med Internet Res.

[26] Escriva Boulley G, Leroy T, Bernetière C, Paquienseguy F, Desfriches-Doria O, Préau M (2018). Digital health interventions to help living with cancer: a systematic review of participants' engagement and psychosocial effects. Psychooncology.

[27] National Academies of Sciences, Engineering, and Medicine (2021). Opportunities and Challenges for Using Digital Health Applications in Oncology: Proceedings of a Workshop.

[28] Peters MGC, McInerney P, Munn Z, Tricco AC, Khalil H (2020). JBI Manual for Evidence Synthesis.

[29] Oh H, Rizo C, Enkin M, Jadad A (2005). What is eHealth (3): a systematic review of published definitions. J Med Internet Res.

[30] (2022). Common cancer types. National Cancer Institute.

[31] (2022). Cancer. World Health Organization.

[32] Unsworth Harriet, Dillon Bernice, Collinson Lucie, Powell Helen, Salmon Mark, Oladapo Tosin, Ayiku Lynda, Shield Gary, Holden Joanne, Patel Neelam, Campbell Mark, Greaves Felix, Joshi Indra, Powell John, Tonnel Alexia (2021). The NICE Evidence Standards Framework for digital health and care technologies - Developing and maintaining an innovative evidence framework with global impact. Digit Health.

[33] Abrahams HJ, Gielissen MFM, Donders RRT, Goedendorp MM, van der Wouw AJ, Verhagen CAHHVM, Knoop H (2017). The efficacy of internet-based cognitive behavioral therapy for severely fatigued survivors of breast cancer compared with care as usual: a randomized controlled trial. Cancer.

[34] Absolom K, Warrington L, Hudson E, Hewison J, Morris C, Holch P, Carter R, Gibson A, Holmes M, Clayton B, Rogers Z, McParland L, Conner M, Glidewell L, Woroncow B, Dawkins B, Dickinson S, Hulme C, Brown J, Velikova G (2021). Phase III randomized controlled trial of eRAPID: eHealth intervention during chemotherapy. J Clin Oncol.

[35] Admiraal JM, van der Velden AWG, Geerling JI, Burgerhof JGM, Bouma G, Walenkamp AME, de Vries EG, Schröder CP, Reyners AK (2017). Web-based tailored psychoeducation for breast cancer patients at the onset of the survivorship phase: a multicenter randomized controlled trial. J Pain Symptom Manage.

[36] Advani P, Brewster AM, Baum GP, Schover LR (2017). A pilot randomized trial to prevent sexual dysfunction in postmenopausal breast cancer survivors starting adjuvant aromatase inhibitor therapy. J Cancer Surviv.

[37] Alberts NM, Leisenring WM, Flynn JS, Whitton J, Gibson TM, Jibb L, McDonald A, Ford J, Moraveji N, Dear BF, Krull KR, Robison LL, Stinson JN, Armstrong GT (2020). Wearable respiratory monitoring and feedback for chronic pain in adult survivors of childhood cancer: a feasibility randomized controlled trial from the childhood cancer survivor study. JCO Clin Cancer Inform.

[38] Allicock M, Kendzor D, Sedory A, Gabriel KP, Swartz MD, Thomas P, Yudkin JS, Rivers A (2021). A pilot and feasibility mobile health intervention to support healthy behaviors in African American breast cancer survivors. J Racial Ethn Health Disparities.

[39] Ariza-Garcia A, Lozano-Lozano M, Galiano-Castillo N, Postigo-Martin P, Arroyo-Morales M, Cantarero-Villanueva I (2019). A web-based exercise system (e-CuidateChemo) to counter the side effects of chemotherapy in patients with breast cancer: randomized controlled trial. J Med Internet Res.

[40] Armstrong KA, Coyte PC, Brown M, Beber B, Semple JL (2017). Effect of home monitoring via mobile app on the number of in-person visits following ambulatory surgery: a randomized clinical trial. JAMA Surg.

[41] Atema V, van Leeuwen M, Kieffer JM, Oldenburg HSA, van Beurden M, Gerritsma MA, Kuenen MA, Plaisier PW, Lopes Cardozo AM, van Riet YE, Heuff G, Rijna H, van der Meij S, Noorda EM, Timmers G, Vrouenraets BC, Bollen M, van der Veen H, Bijker N, Hunter MS, Aaronson NK (2019). Efficacy of internet-based cognitive behavioral therapy for treatment-induced menopausal symptoms in breast cancer survivors: results of a randomized controlled trial. J Clin Oncol.

[42] Bade BC, Gan G, Li F, Lu L, Tanoue L, Silvestri GA, Irwin ML (2021). "Randomized trial of physical activity on quality of life and lung cancer biomarkers in patients with advanced stage lung cancer: a pilot study". BMC Cancer.

[43] Badger T, Segrin C, Pasvogel A, Lopez AM (2013). The effect of psychosocial interventions delivered by telephone and videophone on quality of life in early-stage breast cancer survivors and their supportive partners. J Telemed Telecare.

[44] Baker TB, Hawkins R, Pingree S, Roberts LJ, McDowell HE, Shaw BR, Serlin R, Dillenburg L, Swoboda CM, Han J, Stewart JA, Carmack-Taylor CL, Salner A, Schlam TR, McTavish F, Gustafson DH (2011). Optimizing eHealth breast cancer interventions: which types of eHealth services are effective?. Transl Behav Med.

[45] Bani Mohammad E, Ahmad M (2019). Virtual reality as a distraction technique for pain and anxiety among patients with breast cancer: a randomized control trial. Pall Supp Care.

[46] Beatty L, Kemp E, Coll JR, Turner J, Butow P, Milne D, Yates P, Lambert S, Wootten A, Yip D, Koczwara B (2019). Finding My Way: results of a multicentre RCT evaluating a web-based self-guided psychosocial intervention for newly diagnosed cancer survivors. Support Care Cancer.

[47] Beatty L, Koczwara B, Wade T (2016). Evaluating the efficacy of a self-guided Web-based CBT intervention for reducing cancer-distress: a randomised controlled trial. Support Care Cancer.

[48] Bellens A, Roelant E, Sabbe B, Peeters M, van Dam PA (2020). A video-game based cognitive training for breast cancer survivors with cognitive impairment: a prospective randomized pilot trial. Breast.

[49] Berry DL, Blumenstein BA, Halpenny B, Wolpin S, Fann JR, Austin-Seymour M, Bush N, Karras BT, Lober WB, McCorkle R (2011). Enhancing patient-provider communication with the electronic self-report assessment for cancer: a randomized trial. J Clin Oncol.

[50] Berry DL, Hong F, Blonquist TM, Halpenny B, Filson CP, Master VA, Sanda MG, Chang P, Chien GW, Jones RA, Krupski TL, Wolpin S, Wilson L, Hayes JH, Trinh Q, Sokoloff M, Somayaji P (2018). Decision support with the personal patient profile-prostate: a multicenter randomized trial. J Urol.

[51] Berry DL, Hong F, Halpenny B, Partridge AH, Fann JR, Wolpin S, Lober WB, Bush NE, Parvathaneni U, Back AL, Amtmann D, Ford R (2014). Electronic self-report assessment for cancer and self-care support: results of a multicenter randomized trial. J Clin Oncol.

[52] Bickell NA, Shah A, Castaldi M, Lewis T, Sickles A, Arora S, Clarke K, Kemeny M, Srinivasan A, Fei K, Franco R, Parides M, Pappas P, McAlearney AS (2018). Caution ahead: research challenges of a randomized controlled trial implemented to improve breast cancer treatment at safety-net hospitals. J Oncol Pract.

[53] Blair CK, Harding E, Wiggins C, Kang H, Schwartz M, Tarnower A, Du R, Kinney AY (2021). A home-based mobile health intervention to replace sedentary time with light physical activity in older cancer survivors: randomized controlled pilot trial. JMIR Cancer.

[54] Boele FW, Klein M, Verdonck-de Leeuw IM, Cuijpers P, Heimans JJ, Snijders TJ, Vos M, Bosma I, Tijssen CC, Reijneveld JC, Dutch Society for Neuro-Oncology (LWNO) (2018). Internet-based guided self-help for glioma patients with depressive symptoms: a randomized controlled trial. J Neurooncol.

[55] Bol N, Smets EM, Rutgers MM, Burgers JA, de Haes HC, Loos EF, van Weert JC (2013). Do videos improve website satisfaction and recall of online cancer-related information in older lung cancer patients?. Patient Educ Couns.

[56] Bol N, Smets EMA, Eddes EH, de Haes JCJM, Loos EF, van Weert JCM (2015). Illustrations enhance older colorectal cancer patients' website satisfaction and recall of online cancer information. Eur J Cancer Care (Engl).

[57] Børøsund E, Cvancarova M, Moore SM, Ekstedt M, Ruland CM (2014). Comparing effects in regular practice of e-communication and web-based self-management support among breast cancer patients: preliminary results from a randomized controlled trial. J Med Internet Res.

[58] Børøsund E, Ehlers SL, Varsi C, Clark MM, Andrykowski MA, Cvancarova M, Solberg Nes L (2020). Results from a randomized controlled trial testing StressProffen; an application-based stress-management intervention for cancer survivors. Cancer Med.

[59] Bruggeman-Everts FZ, Wolvers MDJ, van de Schoot R, Vollenbroek-Hutten MMR, Van der Lee ML (2017). Effectiveness of two web-based interventions for chronic cancer-related fatigue compared to an active control condition: results of the "Fitter na kanker" randomized controlled trial. J Med Internet Res.

[60] Bryant AL, Coffman E, Phillips B, Tan X, Bullard E, Hirschey R, Bradley J, Bennett AV, Stover AM, Song L, Shea TC, Wood WA (2020). Pilot randomized trial of an electronic symptom monitoring and reporting intervention for hospitalized adults undergoing hematopoietic stem cell transplantation. Support Care Cancer.

[61] Cadmus-Bertram L, Tevaarwerk AJ, Sesto ME, Gangnon R, Van Remortel B, Date P (2019). Building a physical activity intervention into clinical care for breast and colorectal cancer survivors in Wisconsin: a randomized controlled pilot trial. J Cancer Surviv.

[62] Carpenter KM, Stoner SA, Schmitz K, McGregor BA, Doorenbos AZ (2014). An online stress management workbook for breast cancer. J Behav Med.

[63] Casillas JN, Schwartz LF, Crespi CM, Ganz PA, Kahn KL, Stuber ML, Bastani R, Alquaddomi F, Estrin DL (2019). The use of mobile technology and peer navigation to promote adolescent and young adult (AYA) cancer survivorship care: results of a randomized controlled trial. J Cancer Surviv.

[64] Chee W, Lee Y, Ji X, Chee E, Im EO (2020). The preliminary efficacy of a technology-based cancer pain management program among Asian American breast cancer survivors. Comput Inform Nurs.

[65] Cheville AL, Moynihan T, Herrin J, Loprinzi C, Kroenke K (2019). Effect of collaborative telerehabilitation on functional impairment and pain among patients with advanced-stage cancer: a randomized clinical trial. JAMA Oncol.

[66] Chow EJ, Doody DR, Di C, Armenian SH, Baker KS, Bricker JB, Gopal AK, Hagen AM, Ketterl TG, Lee SJ, Reding KW, Schenk JM, Syrjala KL, Taylor SA, Wang G, Neuhouser ML, Mendoza JA (2021). Feasibility of a behavioral intervention using mobile health applications to reduce cardiovascular risk factors in cancer survivors: a pilot randomized controlled trial. J Cancer Surviv.

[67] Chung IY, Kang E, Yom CK, Kim D, Sun Y, Hwang Y, Jang JY, Kim S (2015). Effect of short message service as a reminder on breast self-examination in breast cancer patients: a randomized controlled trial. J Telemed Telecare.

[68] Çınar D, Karadakovan A, Erdoğan AP (2021). Effect of mobile phone app-based training on the quality of life for women with breast cancer. Eur J Oncol Nurs.

[69] Classen CC, Chivers ML, Urowitz S, Barbera L, Wiljer D, O'Rinn S, Ferguson SE (2013). Psychosexual distress in women with gynecologic cancer: a feasibility study of an online support group. Psychooncology.

[70] Cleeland CS, Wang XS, Shi Q, Mendoza TR, Wright SL, Berry MD, Malveaux D, Shah PK, Gning I, Hofstetter WL, Putnam JB, Vaporciyan AA (2011). Automated symptom alerts reduce postoperative symptom severity after cancer surgery: a randomized controlled clinical trial. J Clin Oncol.

[71] Compen F, Adang E, Bisseling E, van der Lee M, Speckens A (2020). Cost-utility of individual internet-based and face-to-face mindfulness-based cognitive therapy compared with treatment as usual in reducing psychological distress in cancer patients. Psychooncology.

[72] Compen F, Bisseling E, Schellekens M, Donders R, Carlson L, van der Lee M, Speckens A (2018). Face-to-face and internet-based mindfulness-based cognitive therapy compared with treatment as usual in reducing psychological distress in patients with cancer: a multicenter randomized controlled trial. J Clin Oncol.

[73] Cuypers M, Lamers RED, Kil PJM, van de Poll-Franse LV, de Vries M (2018). Impact of a web-based prostate cancer treatment decision aid on patient-reported decision process parameters: results from the Prostate Cancer Patient Centered Care trial. Support Care Cancer.

[74] Darnall B, Ziadni M, Krishnamurthy P, Flood P, Heathcote L, Mackey IG, Taub CJ, Wheeler A (2019). "My surgical success": effect of a digital behavioral pain medicine intervention on time to opioid cessation after breast cancer surgery-a pilot randomized controlled clinical trial. Pain Med.

[75] David N, Schlenker P, Prudlo U, Larbig W (2013). Internet-based program for coping with cancer: a randomized controlled trial with hematologic cancer patients. Psychooncology.

[76] de Hosson LD, Bouma G, Stelwagen J, van Essen H, de Bock GH, de Groot DJA, de Vries EG, Walenkamp AM (2019). Web-based personalised information and support for patients with a neuroendocrine tumour: randomised controlled trial. Orphanet J Rare Dis.

[77] Devine KA, Viola A, Levonyan-Radloff K, Mackowski N, Bozzini B, Chandler A, Xu B, Ohman-Strickland P, Mayans S, Farrar-Anton A, Sahler OJ, Masterson M, Manne S, Arent S (2020). Feasibility of FitSurvivor: a technology-enhanced group-based fitness intervention for adolescent and young adult survivors of childhood cancer. Pediatr Blood Cancer.

[78] Diefenbach MA, Benedict C, Miller SM, Stanton AL, Ropka ME, Wen KY, Fleisher LG, Mohamed NE, Hall SJ (2018). Examining the impact of a multimedia intervention on treatment decision-making among newly diagnosed prostate cancer patients: results from a nationwide RCT. Transl Behav Med.

[79] Dirkse D, Hadjistavropoulos HD, Alberts NA, Karin E, Schneider LH, Titov N, Dear B (2020). Making internet-delivered cognitive behaviour therapy scalable for cancer survivors: a randomized non-inferiority trial of self-guided and technician-guided therapy. J Cancer Surviv.

[80] Dong X, Yi X, Gao D, Gao Z, Huang S, Chao M, Chen W, Ding M (2019). The effects of the combined exercise intervention based on internet and social media software (CEIBISMS) on quality of life, muscle strength and cardiorespiratory capacity in Chinese postoperative breast cancer patients: a randomized controlled trial. Health Qual Life Outcomes.

[81] Donovan HS, Ward SE, Sereika SM, Knapp JE, Sherwood PR, Bender CM, Edwards RP, Fields M, Ingel R (2014). Web-based symptom management for women with recurrent ovarian cancer: a pilot randomized controlled trial of the WRITE Symptoms intervention. J Pain Symptom Manage.

[82] Dos Santos M, Hardy-Léger I, Rigal O, Licaj I, Dauchy S, Levy C, Noal S, Segura C, Delcambre C, Allouache D, Parzy A, Barriere J, Petit T, Lange M, Capel A, Clarisse B, Grellard JM, Lefel J, Joly F (2020). Cognitive rehabilitation program to improve cognition of cancer patients treated with chemotherapy: a 3-arm randomized trial. Cancer.

[83] Duffecy J, Sanford S, Wagner L, Begale M, Nawacki E, Mohr DC (2013). Project onward: an innovative e-health intervention for cancer survivors. Psychooncology.

[84] Ehrbar V, Germeyer A, Nawroth F, Dangel A, Findeklee S, Urech C, Rochlitz C, Stiller R, Tschudin S (2021). Long-term effectiveness of an online decision aid for female cancer patients regarding fertility preservation: knowledge, attitude, and decisional regret. Acta Obstet Gynecol Scand.

[85] Emmons KM, Puleo E, Sprunck-Harrild K, Ford J, Ostroff JS, Hodgson D, Greenberg M, Diller L, de Moor J, Tyc V (2013). Partnership for health-2, a web-based versus print smoking cessation intervention for childhood and young adult cancer survivors: randomized comparative effectiveness study. J Med Internet Res.

[86] Fann JR, Hong F, Halpenny B, Blonquist TM, Berry DL (2017). Psychosocial outcomes of an electronic self-report assessment and self-care intervention for patients with cancer: a randomized controlled trial. Psychooncology.

[87] Ferguson RJ, Sigmon ST, Pritchard AJ, LaBrie SL, Goetze RE, Fink CM, Garrett AM (2016). A randomized trial of videoconference-delivered cognitive behavioral therapy for survivors of breast cancer with self-reported cognitive dysfunction. Cancer.

[88] Ferrante JM, Devine KA, Bator A, Rodgers A, Ohman-Strickland PA, Bandera EV, Hwang KO (2020). Feasibility and potential efficacy of commercial mHealth/eHealth tools for weight loss in African American breast cancer survivors: pilot randomized controlled trial. Transl Behav Med.

[89] Finlay A, Evans H, Vincent A, Wittert G, Vandelanotte C, Short CE (2020). Optimising web-based computer-tailored physical activity interventions for prostate cancer survivors: a randomised controlled trial examining the impact of website architecture on user engagement. Int J Environ Res Public Health.

[90] Fjell M, Langius-Eklöf A, Nilsson M, Wengström Y, Sundberg K (2020). Reduced symptom burden with the support of an interactive app during neoadjuvant chemotherapy for breast cancer - a randomized controlled trial. Breast.

[91] Foley NM, O'Connell EP, Lehane EA, Livingstone V, Maher B, Kaimkhani S, Cil T, Relihan N, Bennett M, Redmond H, Corrigan M (2016). PATI: patient accessed tailored information: a pilot study to evaluate the effect on preoperative breast cancer patients of information delivered via a mobile application. Breast.

[92] Forbes CC, Blanchard CM, Mummery WK, Courneya KS (2017). A pilot study on the motivational effects of an internet-delivered physical activity behaviour change programme in Nova Scotian cancer survivors. Psychol Health.

[93] Fox RS, Moreno PI, Yanez B, Estabrook R, Thomas J, Bouchard LC, McGinty HL, Mohr DC, Begale MJ, Flury SC, Perry KT, Kundu SD, Penedo FJ (2019). Integrating PROMIS® computerized adaptive tests into a web-based intervention for prostate cancer. Health Psychol.

[94] Freeman LW, White R, Ratcliff CG, Sutton S, Stewart M, Palmer JL, Link J, Cohen L (2015). A randomized trial comparing live and telemedicine deliveries of an imagery-based behavioral intervention for breast cancer survivors: reducing symptoms and barriers to care. Psychooncology.

[95] Galiano-Castillo N, Arroyo-Morales M, Lozano-Lozano M, Fernández-Lao C, Martín-Martín L, Del-Moral-Ávila R, Cantarero-Villanueva I (2017). Effect of an internet-based telehealth system on functional capacity and cognition in breast cancer survivors: a secondary analysis of a randomized controlled trial. Support Care Cancer.

[96] Galiano-Castillo N, Cantarero-Villanueva I, Fernández-Lao C, Ariza-García A, Díaz-Rodríguez L, Del-Moral-Ávila R, Arroyo-Morales M (2016). Telehealth system: a randomized controlled trial evaluating the impact of an internet-based exercise intervention on quality of life, pain, muscle strength, and fatigue in breast cancer survivors. Cancer.

[97] Galvão DA, Newton RU, Girgis A, Lepore SJ, Stiller A, Mihalopoulos C, Gardiner RA, Taaffe DR, Occhipinti S, Chambers SK (2018). Randomized controlled trial of a peer led multimodal intervention for men with prostate cancer to increase exercise participation. Psychooncology.

[98] Gehring K, Kloek CJ, Aaronson NK, Janssen KW, Jones LW, Sitskoorn MM, Stuiver MM (2018). Feasibility of a home-based exercise intervention with remote guidance for patients with stable grade II and III gliomas: a pilot randomized controlled trial. Clin Rehabil.

[99] Gell NM, Grover KW, Savard L, Dittus K (2020). Outcomes of a text message, Fitbit, and coaching intervention on physical activity maintenance among cancer survivors: a randomized control pilot trial. J Cancer Surviv.

[100] Ghanbari E, Yektatalab S, Mehrabi M (2021). Effects of psychoeducational interventions using mobile apps and mobile-based online group discussions on anxiety and self-esteem in women with breast cancer: randomized controlled trial. JMIR Mhealth Uhealth.

[101] Giesler JM, Keller B, Repke T, Leonhart R, Weis J, Muckelbauer R, Rieckmann N, Müller-Nordhorn J, Lucius-Hoene G, Holmberg C (2017). Effect of a website that presents patients' experiences on self-efficacy and patient competence of colorectal cancer patients: web-based randomized controlled trial. J Med Internet Res.

[102] Gnagnarella P, Misotti AM, Santoro L, Akoumianakis D, Del Campo L, De Lorenzo F, Lombardo C, Milolidakis G, Sullivan R, McVie JG (2016). Nutritional online information for cancer patients: a randomized trial of an internet communication plus social media intervention. J Cancer Educ.

[103] Golsteijn RHJ, Bolman C, Volders E, Peels DA, de Vries H, Lechner L (2018). Short-term efficacy of a computer-tailored physical activity intervention for prostate and colorectal cancer patients and survivors: a randomized controlled trial. Int J Behav Nutr Phys Act.

[104] Gomersall SR, Skinner TL, Winkler E, Healy GN, Eakin E, Fjeldsoe B (2019). Feasibility, acceptability and efficacy of a text message-enhanced clinical exercise rehabilitation intervention for increasing 'whole-of-day' activity in people living with and beyond cancer. BMC Public Health.

[105] Gornick MC, Kurian AW, An LC, Fagerlin A, Jagsi R, Katz SJ, Hawley ST (2018). Knowledge regarding and patterns of genetic testing in patients newly diagnosed with breast cancer participating in the iCanDecide trial. Cancer.

[106] Graetz I, McKillop CN, Stepanski E, Vidal GA, Anderson JN, Schwartzberg LS (2018). Use of a web-based app to improve breast cancer symptom management and adherence for aromatase inhibitors: a randomized controlled feasibility trial. J Cancer Surviv.

[107] Greer JA, Jacobs J, Pensak N, MacDonald JJ, Fuh CX, Perez GK, Ward A, Tallen C, Muzikansky A, Traeger L, Penedo FJ, El-Jawahri A, Safren SA, Pirl WF, Temel JS (2019). Randomized trial of a tailored cognitive-behavioral therapy mobile application for anxiety in patients with incurable cancer. Oncologist.

[108] Greer Joseph A, Jacobs Jamie M, Pensak Nicole, Nisotel Lauren E, Fishbein Joel N, MacDonald James J, Ream Molly E, Walsh Emily A, Buzaglo Joanne, Muzikansky Alona, Lennes Inga T, Safren Steven A, Pirl William F, Temel Jennifer S (2020). Randomized Trial of a Smartphone Mobile App to Improve Symptoms and Adherence to Oral Therapy for Cancer. J Natl Compr Canc Netw.

[109] Greer S, Ramo D, Chang Y, Fu M, Moskowitz J, Haritatos J (2019). Use of the chatbot "Vivibot" to deliver positive psychology skills and promote well-being among young people after cancer treatment: randomized controlled feasibility trial. JMIR Mhealth Uhealth.

[110] Gustafson DH, DuBenske LL, Namkoong K, Hawkins R, Chih M, Atwood AK, Johnson R, Bhattacharya A, Carmack CL, Traynor AM, Campbell TC, Buss MK, Govindan R, Schiller JH, Cleary JF (2013). An eHealth system supporting palliative care for patients with non-small cell lung cancer: a randomized trial. Cancer.

[111] Haggerty AF, Huepenbecker S, Sarwer DB, Spitzer J, Raggio G, Chu CS, Ko E, Allison KC (2016). The use of novel technology-based weight loss interventions for obese women with endometrial hyperplasia and cancer. Gynecol Oncol.

[112] Handa S, Okuyama H, Yamamoto H, Nakamura S, Kato Y (2020). Effectiveness of a smartphone application as a support tool for patients undergoing breast cancer chemotherapy: a randomized controlled trial. Clin Breast Cancer.

[113] Hardcastle SJ, Maxwell-Smith C, Hince D, Bulsara MK, Boyle T, Tan P, Levitt M, Salama P, Mohan GR, Salfinger S, Makin G, Tan J, Platell C, Cohen PA (2021). The wearable activity technology and action-planning trial in cancer survivors: physical activity maintenance post-intervention. J Sci Med Sport.

[114] Hawkins RP, Pingree S, Baker TB, Roberts LJ, Shaw BR, McDowell H, Serlin RC, Dillenburg L, Swoboda CM, Han J, Stewart JA, Carmack CL, Salner A, Schlam TR, McTavish F, Gustafson DH (2011). Integrating eHealth with human services for breast cancer patients. Transl Behav Med.

[115] Hawkins RP, Pingree S, Van Bogaert D, McDowell H, Jarrard D, Carmack C, Salner A (2017). The impact of combining human and online supportive resources for prostate cancer patients. J Community Supportive Oncol.

[116] Hershman DL, Unger JM, Hillyer GC, Moseley A, Arnold KB, Dakhil SR, Esparaz BT, Kuan MC, Graham ML, Lackowski DM, Edenfield WJ, Dayao ZR, Henry NL, Gralow JR, Ramsey SD, Neugut AI (2020). Randomized trial of text messaging to reduce early discontinuation of adjuvant aromatase inhibitor therapy in women with early-stage breast cancer: SWOG S1105. J Clin Oncol.

[117] Holtdirk F, Mehnert A, Weiss M, Mayer J, Meyer BR, Bröde P, Claus M, Watzl C (2021). Results of the Optimune trial: a randomized controlled trial evaluating a novel internet intervention for breast cancer survivors. PLoS One.

[118] Hou IC, Lin HY, Shen SH, Chang KJ, Tai HC, Tsai AJ, Dykes PC (2020). Quality of life of women after a first diagnosis of breast cancer using a self-management support mHealth app in Taiwan: randomized controlled trial. JMIR Mhealth Uhealth.

[119] Høybye M T, Dalton SO, Deltour I, Bidstrup PE, Frederiksen K, Johansen C (2010). Effect of internet peer-support groups on psychosocial adjustment to cancer: a randomised study. Br J Cancer.

[120] Huang CC, Kuo HP, Lin YE, Chen SC (2019). Effects of a web-based health education program on quality of life and symptom distress of initially diagnosed advanced non-small cell lung cancer patients: a randomized controlled trial. J Cancer Educ.

[121] Im EO, Kim S, Lee C, Chee E, Mao JJ, Chee W (2019). Decreasing menopausal symptoms of Asian American breast cancer survivors through a technology-based information and coaching/support program. Menopause.

[122] Irene Su H, Stark S, Kwan B, Boles S, Chingos D, Ehren J, Gorman JR, Krychman M, Romero SA, Mao JJ, Pierce JP, Natarajan L (2019). Efficacy of a web-based women's health survivorship care plan for young breast cancer survivors: a randomized controlled trial. Breast Cancer Res Treat.

[123] Ji W, Kwon H, Lee S, Kim S, Hong JS, Park YR, Kim HR, Lee JC, Jung EJ, Kim D, Choi C (2019). Mobile health management platform-based pulmonary rehabilitation for patients with non-small cell lung cancer: prospective clinical trial. JMIR Mhealth Uhealth.

[124] Jim HSL, Hyland KA, Nelson AM, Pinilla-Ibarz J, Sweet K, Gielissen M, Bulls H, Hoogland AI, Jacobsen PB, Knoop H (2020). Internet-assisted cognitive behavioral intervention for targeted therapy-related fatigue in chronic myeloid leukemia: results from a pilot randomized trial. Cancer.

[125] Jolly TA, Deal AM, Mariano C, Markowski N, Kirk S, Perlmutt MS, Jones F, Choi SK, Nyrop KA, Busby-Whitehead J, Muss H (2020). A randomized trial of real-time geriatric assessment reporting in nonelectively hospitalized older adults with cancer. Oncologist.

[126] Joo SC, Navidian A, Sharifi S (2020). Evaluating the effectiveness of planned discharge program in the quality of life of gastrointestinal cancer patients undergoing chemotherapy: a clinical trial study. Med Surg Nurs J.

[127] Kanera IM, Bolman CA, Willems RA, Mesters I, Lechner L (2016). Lifestyle-related effects of the web-based Kanker Nazorg Wijzer (Cancer Aftercare Guide) intervention for cancer survivors: a randomized controlled trial. J Cancer Surviv.

[128] Kanera IM, Willems RA, Bolman CAW, Mesters I, Verboon P, Lechner L (2017). Long-term effects of a web-based cancer aftercare intervention on moderate physical activity and vegetable consumption among early cancer survivors: a randomized controlled trial. Int J Behav Nutr Phys Act.

[129] Kearney N, McCann L, Norrie J, Taylor L, Gray P, McGee-Lennon M, Sage M, Miller M, Maguire R (2009). Evaluation of a mobile phone-based, advanced symptom management system (ASyMS) in the management of chemotherapy-related toxicity. Support Care Cancer.

[130] Kekäle M, Söderlund T, Koskenvesa P, Talvensaari K, Airaksinen M (2016). Impact of tailored patient education on adherence of patients with chronic myeloid leukaemia to tyrosine kinase inhibitors: a randomized multicentre intervention study. J Adv Nurs.

[131] Kenfield SA, Van Blarigan EL, Ameli N, Lavaki E, Cedars B, Paciorek AT, Monroy C, Tantum LK, Newton RU, Signorell C, Suh JH, Zhang L, Cooperberg MR, Carroll PR, Chan JM (2019). Feasibility, acceptability, and behavioral outcomes from a technology-enhanced behavioral change intervention (prostate 8): a pilot randomized controlled trial in men with prostate cancer. Eur Urol.

[132] Kesler S, Hadi Hosseini SM, Heckler C, Janelsins M, Palesh O, Mustian K, Morrow G (2013). Cognitive training for improving executive function in chemotherapy-treated breast cancer survivors. Clin Breast Cancer.

[133] Kim HJ, Kim SM, Shin H, Jang JS, Kim YI, Han DH (2018). A mobile game for patients with breast cancer for chemotherapy self-management and quality-of-life improvement: randomized controlled trial. J Med Internet Res.

[134] Kim SC, Hawkins RP, Shah DV, Gustafson DH, Baker TB (2020). Understanding how e-health interventions meet psychosocial needs of breast cancer patients: the pathways of influence on quality of life and cancer concerns. Psychooncology.

[135] Knoerl R, Smith EML, Barton DL, Williams DA, Holden JE, Krauss JC, LaVasseur B (2018). Self-guided online cognitive behavioral strategies for chemotherapy-induced peripheral neuropathy: a multicenter, pilot, randomized, wait-list controlled trial. J Pain.

[136] Kolb NA, Smith AG, Singleton JR, Beck SL, Howard D, Dittus K, Karafiath S, Mooney K (2018). Chemotherapy-related neuropathic symptom management: a randomized trial of an automated symptom-monitoring system paired with nurse practitioner follow-up. Support Care Cancer.

[137] Kong S, Lee JK, Kang D, Kim N, Shim YM, Park W, Choi D, Cho J (2021). Comparing the effectiveness of a wearable activity tracker in addition to counseling and counseling only to reinforce leisure-time physical activity among breast cancer patients: a randomized controlled trial. Cancers (Basel).

[138] Korkmaz S, Iyigun E, Tastan S (2020). An evaluation of the influence of web-based patient education on the anxiety and life quality of patients who have undergone mammaplasty: a randomized controlled study. J Cancer Educ.

[139] Krebs P, Burkhalter J, Fiske J, Snow H, Schofield E, Iocolano M, Borderud S, Ostroff JS (2019). The QuitIT coping skills game for promoting tobacco cessation among smokers diagnosed with cancer: pilot randomized controlled trial. JMIR Mhealth Uhealth.

[140] Kroenke K, Theobald D, Wu J, Norton K, Morrison G, Carpenter J, Tu W (2010). Effect of telecare management on pain and depression in patients with cancer: a randomized trial. JAMA.

[141] Kubo A, Kurtovich E, McGinnis M, Aghaee S, Altschuler A, Quesenberry C, Kolevska T, Avins AL (2019). A randomized controlled trial of mHealth mindfulness intervention for cancer patients and informal cancer caregivers: a feasibility study within an integrated health care delivery system. Integr Cancer Ther.

[142] Lally RM, Kupzyk KA, Bellavia G, Hydeman J, Gallo S, Helgeson VS, Erwin D, Mills AC, Brown JK (2020). CaringGuidance™ after breast cancer diagnosis eHealth psychoeducational intervention to reduce early post-diagnosis distress. Support Care Cancer.

[143] Lee BJ, Park YH, Lee JY, Kim SJ, Jang Y, Lee JI (2019). Smartphone application versus pedometer to promote physical activity in prostate cancer patients. Telemed J E Health.

[144] Lepore SJ, Buzaglo JS, Lieberman MA, Golant M, Greener JR, Davey A (2014). Comparing standard versus prosocial internet support groups for patients with breast cancer: a randomized controlled trial of the helper therapy principle. J Clin Oncol.

[145] Li L, Wang L, Sun Q, Xiao P, Duan Y, Liu X, Zhou J, Xie J, Cheng AS (2022). Effect of two interventions on sleep quality for adolescent and young adult cancer survivors: a pilot randomized controlled trial. Cancer Nurs.

[146] Livingston PM, Heckel L, Orellana L, Ashley D, Ugalde A, Botti M, Pitson G, Woollett A, Chambers SK, Parente P, Chirgwin J, Mihalopoulos C, Lavelle B, Sutton J, Phipps-Nelson J, Krishnasamy M, Simons K, Heynsbergh N, Wickramasinghe N, White V (2020). Outcomes of a randomized controlled trial assessing a smartphone Application to reduce unmet needs among people diagnosed with CancEr (ACE). Cancer Med.

[147] Lleras de Frutos M, Medina JC, Vives J, Casellas-Grau A, Marzo JL, Borràs JM, Ochoa-Arnedo C (2020). Video conference vs face-to-face group psychotherapy for distressed cancer survivors: a randomized controlled trial. Psychooncology.

[148] Longacre CF, Nyman JA, Visscher SL, Borah BJ, Cheville AL (2020). Cost-effectiveness of the Collaborative Care to Preserve Performance in Cancer (COPE) trial tele-rehabilitation interventions for patients with advanced cancers. Cancer Med.

[149] Lozano-Lozano M, Martín-Martín L, Galiano-Castillo N, Fernández-Lao C, Cantarero-Villanueva I, López-Barajas IB, Arroyo-Morales M (2020). Mobile health and supervised rehabilitation versus mobile health alone in breast cancer survivors: randomized controlled trial. Ann Phys Rehabil Med.

[150] Lynch BM, Nguyen NH, Moore MM, Reeves MM, Rosenberg DE, Boyle T, Vallance JK, Milton S, Friedenreich CM, English DR (2019). A randomized controlled trial of a wearable technology-based intervention for increasing moderate to vigorous physical activity and reducing sedentary behavior in breast cancer survivors: the ACTIVATE Trial. Cancer.

[151] Lynch C, Bird S, Barnett F, Lythgo N, Selva-Raj I (2021). Improving the physical activity of breast cancer survivors through fitness trackers. Studies in Health Technology and Informatics.

[152] Lyu KX, Zhao J, Wang B, Xiong GX, Yang WQ, Liu QH, Zhu X, Sun W, Jiang A, Wen W, Lei W (2016). Smartphone application WeChat for clinical follow-up of discharged patients with head and neck tumors: a randomized controlled trial. Chin Med J (Engl).

[153] Manne SL, Topham N, D'Agostino TA, Myers Virtue S, Kirstein L, Brill K, Manning C, Grana G, Schwartz MD, Ohman-Strickland P (2016). Acceptability and pilot efficacy trial of a web-based breast reconstruction decision support aid for women considering mastectomy. Psychooncology.

[154] Maxwell-Smith C, Hince D, Cohen PA, Bulsara MK, Boyle T, Platell C, Tan P, Levitt M, Salama P, Tan J, Salfinger S, Makin G, Mohan GR, Jiménez-Castuera R, Hardcastle SJ (2019). A randomized controlled trial of WATAAP to promote physical activity in colorectal and endometrial cancer survivors. Psychooncology.

[155] Mayer DK, Landucci G, Awoyinka L, Atwood AK, Carmack CL, Demark-Wahnefried W, McTavish F, Gustafson DH (2018). SurvivorCHESS to increase physical activity in colon cancer survivors: can we get them moving?. J Cancer Surviv.

[156] Meropol NJ, Egleston BL, Buzaglo JS, Balshem A, Benson AB, Cegala DJ, Cohen RB, Collins M, Diefenbach MA, Miller SM, Fleisher L, Millard JL, Ross EA, Schulman KA, Silver A, Slater E, Solarino N, Sulmasy DP, Trinastic J, Weinfurt KP (2013). A Web-based communication aid for patients with cancer: the CONNECT Study. Cancer.

[157] Meropol NJ, Wong YN, Albrecht T, Manne S, Miller SM, Flamm AL, Benson AB, Buzaglo J, Collins M, Egleston B, Fleisher L, Katz M, Kinzy TG, Liu TM, Margevicius S, Miller DM, Poole D, Roach N, Ross E, Schluchter MD (2016). Randomized trial of a web-based intervention to address barriers to clinical trials. J Clin Oncol.

[158] Mihuta ME, Green HJ, Shum DHK (2018). Web-based cognitive rehabilitation for survivors of adult cancer: a randomised controlled trial. Psychooncology.

[159] Milbury K, Weathers SP, Durrani S, Li Y, Whisenant M, Li J, Lim B, Weinberg JS, Kesler SR, Cohen L, Bruera E (2020). Online couple-based meditation intervention for patients with primary or metastatic brain tumors and their partners: results of a pilot randomized controlled trial. J Pain Symptom Manage.

[160] Millstine DM, Bhagra A, Jenkins SM, Croghan IT, Stan DL, Boughey JC, Nguyen MT, Pruthi S (2019). Use of a wearable EEG headband as a meditation device for women with newly diagnosed breast cancer: a randomized controlled trial. Integr Cancer Ther.

[161] Mooney KH, Beck SL, Friedman RH, Farzanfar R, Wong B (2014). Automated monitoring of symptoms during ambulatory chemotherapy and oncology providers' use of the information: a randomized controlled clinical trial. Support Care Cancer.

[162] Mooney KH, Beck SL, Wong B, Dunson W, Wujcik D, Whisenant M, Donaldson G (2017). Automated home monitoring and management of patient-reported symptoms during chemotherapy: results of the symptom care at home RCT. Cancer Med.

[163] Murphy MJ, Newby JM, Butow P, Loughnan SA, Joubert AE, Kirsten L, Allison K, Shaw J, Shepherd H, Smith J, Andrews G (2020). Randomised controlled trial of internet-delivered cognitive behaviour therapy for clinical depression and/or anxiety in cancer survivors (iCanADAPT Early). Psychooncology.

[164] Nguyen MH, Smets EM, Bol N, Loos EF, van Laarhoven HW, Geijsen D, van Berge Henegouwen MI, Tytgat KM, van Weert JC (2019). Tailored web-based information for younger and older patients with cancer: randomized controlled trial of a preparatory educational intervention on patient outcomes. J Med Internet Res.

[165] Nguyen NH, Vallance JK, Buman MP, Moore MM, Reeves MM, Rosenberg DE, Boyle T, Milton S, Friedenreich CM, English DR, Lynch BM (2021). Effects of a wearable technology-based physical activity intervention on sleep quality in breast cancer survivors: the ACTIVATE Trial. J Cancer Surviv.

[166] Nicolaije KAH, Ezendam NPM, Vos MC, Pijnenborg JMA, Boll D, Boss EA, Hermans RH, Engelhart KC, Haartsen JE, Pijlman BM, van Loon-Baelemans IE, Mertens HJ, Nolting WE, van Beek JJ, Roukema JA, Zijlstra WP, Kruitwagen RF, van de Poll-Franse LV (2015). Impact of an automatically generated cancer survivorship care plan on patient-reported outcomes in routine clinical practice: longitudinal outcomes of a pragmatic, cluster randomized trial. J Clin Oncol.

[167] Ormel HL, van der Schoot GGF, Westerink NDL, Sluiter WJ, Gietema JA, Walenkamp AM (2018). Self-monitoring physical activity with a smartphone application in cancer patients: a randomized feasibility study (SMART-trial). Support Care Cancer.

[168] Osei DK, Lee JW, Modest NN, Pothier PK (2013). Effects of an online support group for prostate cancer survivors: a randomized trial. Soc Urol Nurse Associates.

[169] Owen JE, Klapow JC, Roth DL, Shuster Jr JL, Bellis J, Meredith R, Tucker DC (2005). Randomized pilot of a self-guided internet coping group for women with early-stage breast cancer. Ann Behav Med.

[170] Owen JE, O'Carroll Bantum E, Pagano IS, Stanton A (2017). Randomized trial of a social networking intervention for cancer-related distress. Ann Behav Med.

[171] Oyama H, Kaneda M, Katsumata N, Akechi T, Ohsuga M (2000). Using the bedside wellness system during chemotherapy decreases fatigue and emesis in cancer patients. J Med Syst.

[172] Penedo FJ, Fox RS, Walsh EA, Yanez B, Miller GE, Oswald LB, Estabrook R, Chatterton RT, Mohr DC, Begale MJ, Flury SC, Perry K, Kundu SD, Moreno PI (2021). Effects of web-based cognitive behavioral stress management and health promotion interventions on neuroendocrine and inflammatory markers in men with advanced prostate cancer: a randomized controlled trial. Brain Behav Immun.

[173] Peng Z, Li L, Chen Y, Feng Z, Fang X (2020). WeChat app-based reinforced education improves the quality of opioid titration treatment of cancer-related pain in outpatients: a randomized control study. BMC Cancer.

[174] Pfeifer MP, Keeney C, Bumpous J, Schapmire TJ, Studts JL, Myers J, Head B (2015). Impact of a telehealth intervention on quality of life and symptom distress in patients with head and neck cancer. J Community Support Oncol.

[175] Politi M, Lee CN, Philpott-Streiff SE, Foraker RE, Olsen MA, Merrill C, Tao Yu, Myckatyn TM (2020). A randomized controlled trial evaluating the BREASTChoice tool for personalized decision support about breast reconstruction after mastectomy. Ann Surg.

[176] Porter LS, Gao X, Lyna P, Kraus W, Olsen M, Patterson E, Puleo B, Pollak KI (2018). Pilot randomized trial of a couple-based physical activity videoconference intervention for sedentary cancer survivors. Health Psychol.

[177] Post DM, Shapiro CL, Cegala DJ, David P, Katz ML, Krok JL, Phillips GS, McAlearney AS, Lehman JS, Hicks W, Paskett ED (2013). Improving symptom communication through personal digital assistants: the CHAT (Communicating Health Assisted by Technology) project. J Natl Cancer Inst Monogr.

[178] Rabin C, Dunsiger S, Ness KK, Marcus BH (2011). Internet-based physical activity intervention targeting young adult cancer survivors. J Adolesc Young Adult Oncol.

[179] Rico TM, Dos Santos Machado K, Fernandes VP, Madruga SW, Santin MM, Petrarca CR, Dumith SC (2020). Use of text messaging (SMS) for the management of side effects in cancer patients undergoing chemotherapy treatment: a randomized controlled trial. J Med Syst.

[180] Ridner SH, Dietrich MS, Davis AJ, Sinclair V (2020). A randomized clinical trial comparing the impact of a web-based multimedia intervention versus an educational pamphlet on patient outcomes in breast cancer survivors with chronic secondary lymphedema. J Womens Health (Larchmt).

[181] Ritterband LM, Bailey ET, Thorndike FP, Lord HR, Farrell-Carnahan L, Baum LD (2012). Initial evaluation of an internet intervention to improve the sleep of cancer survivors with insomnia. Psychooncology.

[182] Rojas EO, Anthony CA, Kain J, Glass N, Shah A, Smith T, Miller BJ (2019). Automated mobile phone messaging utilizing a cognitive behavioral intervention: a pilot investigation. Iowa Orthop J.

[183] Ruland CM, Andersen T, Jeneson A, Moore S, Grimsbø GH, Børøsund E, Ellison MC (2013). Effects of an internet support system to assist cancer patients in reducing symptom distress: a randomized controlled trial. Cancer Nurs.

[184] Ruland CM, Holte HH, Røislien J, Heaven C, Hamilton GA, Kristiansen J, Sandbaek H, Kvaløy SO, Hasund L, Ellison MC (2010). Effects of a computer-supported interactive tailored patient assessment tool on patient care, symptom distress, and patients' need for symptom management support: a randomized clinical trial. J Am Med Inform Assoc.

[185] Russell L, Ugalde A, Orellana L, Milne D, Krishnasamy M, Chambers R, Austin DW, Livingston PM (2019). A pilot randomised controlled trial of an online mindfulness-based program for people diagnosed with melanoma. Support Care Cancer.

[186] Ryhänen AM, Rankinen S, Siekkinen M, Saarinen M, Korvenranta H, Leino-Kilpi H (2012). The impact of an empowering Internet-based Breast Cancer Patient Pathway programme on breast cancer patients' knowledge: a randomised control trial. Patient Educ Couns.

[187] Salzer MS, Palmer SC, Kaplan K, Brusilovskiy E, Ten Have T, Hampshire M, Metz J, Coyne JC (2010). A randomized, controlled study of Internet peer-to-peer interactions among women newly diagnosed with breast cancer. Psychooncology.

[188] Sansom-Daly UM, Wakefield CE, Ellis SJ, McGill BC, Donoghoe MW, Butow P, Bryant R, Sawyer S, Patterson P, Anazodo A, Plaster M, Thompson K, Holland L, Osborn M, Maguire F, O'Dwyer C, De Abreu Lourenco R, Cohn R (2021). Online, group-based psychological support for adolescent and young adult cancer survivors: results from the recapture life randomized trial. Cancers (Basel).

[189] Schover LR, Yuan Y, Fellman BM, Odensky E, Lewis PE, Martinetti P (2013). Efficacy trial of an Internet-based intervention for cancer-related female sexual dysfunction. J Natl Compr Canc Netw.

[190] Schwartz AL, Biddle-Newberry M, de Heer HD (2015). Randomized trial of exercise and an online recovery tool to improve rehabilitation outcomes of cancer survivors. Phys Sportsmed.

[191] Schwartz LA, Daniel LC, Henry-Moss D, Bonafide CP, Li Y, Psihogios AM, Butler ES, Szalda D, Ver Hoeve ES, Hobbie WL, Dowshen NL, Pierce L, Kersun LS, Barakat LP (2020). Feasibility and acceptability of a pilot tailored text messaging intervention for adolescents and young adults completing cancer treatment. Psychooncology.

[192] Schwenk M, Grewal GS, Holloway D, Muchna A, Garland L, Najafi B (2016). Interactive sensor-based balance training in older cancer patients with chemotherapy-induced peripheral neuropathy: a randomized controlled trial. Gerontology.

[193] Sherman KA, Przezdziecki A, Alcorso J, Kilby CJ, Elder E, Boyages J, Koelmeyer L, Mackie H (2018). Reducing body image-related distress in women with breast cancer using a structured online writing exercise: results from the my changed body randomized controlled trial. J Clin Oncol.

[194] Siekkinen M, Kesänen J, Vahlberg T, Pyrhönen S, Leino-Kilpi H (2015). Randomized, controlled trial of the effect of e-feedback on knowledge about radiotherapy of breast cancer patients in Finland. Nurs Health Sci.

[195] Siekkinen M, Pyrhönen S, Ryhänen A, Vahlberg T, Leino-Kilpi H (2015). Psychosocial outcomes of e-feedback of radiotherapy for breast cancer patients: a randomized controlled trial. Psychooncology.

[196] Smith SK, MacDermott K, Amarasekara S, Pan W, Mayer D, Hockenberry M (2019). Reimagine: a randomized controlled trial of an online, symptom self-management curriculum among breast cancer survivors. Support Care Cancer.

[197] Smith SK, Westbrook K, MacDermott K, Amarasekara S, LeBlanc M, Pan W (2020). Four conversations: a randomized controlled trial of an online, personalized coping and decision aid for metastatic breast cancer patients. J Palliat Med.

[198] Song L, Guo P, Tan X, Chen RC, Nielsen ME, Birken SA, Koontz BF, Northouse LL, Mayer DK (2021). Enhancing survivorship care planning for patients with localized prostate cancer using a couple-focused web-based, mHealth program: the results of a pilot feasibility study. J Cancer Surviv.

[199] Spahrkäs SS, Looijmans A, Sanderman R, Hagedoorn M (2020). Beating cancer-related fatigue with the Untire mobile app: results from a waiting-list randomized controlled trial. Psychooncology.

[200] Spoelstra SL, Given BA, Given CW, Grant M, Sikorskii A, You M, Decker V (2013). An intervention to improve adherence and management of symptoms for patients prescribed oral chemotherapy agents: an exploratory study. Cancer Nurs.

[201] Spoelstra SL, Given CW, Sikorskii A, Coursaris CK, Majumder A, DeKoekkoek T, Schueller M, Given B (2015). Feasibility of a text messaging intervention to promote self-management for patients prescribed oral anticancer agents. Oncol Nursing forum.

[202] Stankowski-Drengler TJ, Tucholka JL, Bruce JG, Steffens NM, Schumacher JR, Greenberg CC, Wilke LG, Hanlon B, Steiman J, Neuman HB (2019). A randomized controlled trial evaluating the impact of pre-consultation information on patients' perception of information conveyed and satisfaction with the decision-making process. Ann Surg Oncol.

[203] Stanton AL, Thompson EH, Crespi CM, Link JS, Waisman JR (2013). Project connect online: randomized trial of an internet-based program to chronicle the cancer experience and facilitate communication. J Clin Oncol.

[204] Steel JL, Geller DA, Kim KH, Butterfield LH, Spring M, Grady J, Sun W, Marsh W, Antoni M, Dew MA, Helgeson V, Schulz R, Tsung A (2016). Web-based collaborative care intervention to manage cancer-related symptoms in the palliative care setting. Cancer.

[205] Stevenson W, Bryant J, Watson R, Sanson-Fisher R, Oldmeadow C, Henskens F, Brown C, Ramanathan S, Tiley C, Enjeti A, Guest J, Tzelepis F, Paul C, D'Este C (2020). A multi-center randomized controlled trial to reduce unmet needs, depression, and anxiety among hematological cancer patients and their support persons. J Psychosoc Oncol.

[206] Sturgeon KM, Dean LT, Heroux M, Kane J, Bauer T, Palmer E, Long J, Lynch S, Jacobs L, Sarwer DB, Leonard MB, Schmitz K (2017). Commercially available lifestyle modification program: randomized controlled trial addressing heart and bone health in BRCA1/2+ breast cancer survivors after risk-reducing salpingo-oophorectomy. J Cancer Surviv.

[207] Sui Y, Wang T, Wang X (2020). The impact of WeChat app-based education and rehabilitation program on anxiety, depression, quality of life, loss of follow-up and survival in non-small cell lung cancer patients who underwent surgical resection. Eur J Oncol Nurs.

[208] Syrjala KL, Yi JC, Artherholt SB, Romano JM, Crouch ML, Fiscalini AS, Hegel MT, Flowers ME, Martin PJ, Leisenring WM (2018). An online randomized controlled trial, with or without problem-solving treatment, for long-term cancer survivors after hematopoietic cell transplantation. J Cancer Surviv.

[209] Tagai EK, Miller SM, Hudson SV, Diefenbach MA, Handorf E, Bator A, Marziliano A, Kutikov A, Hall SJ, Vira M, Schwartz M, Kim IY, Kim S (2021). Improved cancer coping from a web-based intervention for prostate cancer survivors: a randomized controlled trial. Psychooncology.

[210] Tan EH, Wong ALA, Tan CC, Wong P, Tan SH, Ang LEY, Lim SE, Chong WQ, Ho J, Lee SC, Tai BC (2020). Improving medication adherence with adjuvant aromatase inhibitor in women with breast cancer: a randomised controlled trial to evaluate the effect of short message service (SMS) reminder. Breast.

[211] Tucholka JL, Yang DY, Bruce JG, Steffens NM, Schumacher JR, Greenberg CC, Wilke LG, Steiman J, Neuman HB (2018). A randomized controlled trial evaluating the impact of web-based information on breast cancer patients' knowledge of surgical treatment options. J Am Coll Surg.

[212] Urech Corinne, Grossert Astrid, Alder J, Scherer Sandra, Handschin Barbara, Kasenda Benjamin, Borislavova Borislava, Degen Sven, Erb Jennifer, Faessler Alexandra, Gattlen Laura, Schibli Sarah, Werndli Celine, Gaab Jens, Berger Thomas, Zumbrunn Thomas, Hess Viviane (2018). Web-Based Stress Management for Newly Diagnosed Patients With Cancer (STREAM): A Randomized, Wait-List Controlled Intervention Study. J Clin Oncol.

[213] Vallance JK, Nguyen NH, Moore MM, Reeves MM, Rosenberg DE, Boyle T, Milton S, Friedenreich CM, English DR, Lynch BM (2020). Effects of the ACTIVity And TEchnology (ACTIVATE) intervention on health-related quality of life and fatigue outcomes in breast cancer survivors. Psychooncology.

[214] Valle CG, Tate DF, Mayer DK, Allicock M, Cai J (2013). A randomized trial of a Facebook-based physical activity intervention for young adult cancer survivors. J Cancer Surviv.

[215] Van Blarigan EL, Kenfield SA, Chan JM, Van Loon K, Paciorek A, Zhang L, Chan H, Savoie MB, Bocobo AG, Liu VN, Wong VX, Laffan A, Atreya CE, Miaskowski C, Fukuoka Y, Meyerhardt JA, Venook AP (2020). Feasibility and acceptability of a web-based dietary intervention with text messages for colorectal cancer: a randomized pilot trial. Cancer Epidemiol Biomarkers Prev.

[216] van Bruinessen IR, van Weel-Baumgarten EM, Gouw H, Zijlstra JM, van Dulmen S (2016). An integrated process and outcome evaluation of a web-based communication tool for patients with malignant lymphoma: randomized controlled trial. J Med Internet Res.

[217] van de Wal M, Thewes B, Gielissen M, Speckens A, Prins J (2017). Efficacy of blended cognitive behavior therapy for high fear of recurrence in breast, prostate, and colorectal cancer survivors: the SWORD study, a randomized controlled trial. J Clin Oncol.

[218] van den Berg SW, Gielissen MFM, Custers JAE, van der Graaf WTA, Ottevanger PB, Prins JB (2015). BREATH: web-based self-management for psychological adjustment after primary breast cancer—results of a multicenter randomized controlled trial. J Clin Oncol.

[219] van der Hout A, Jansen F, van Uden-Kraan CF, Coupé VM, Holtmaat K, Nieuwenhuijzen GA, Hardillo JA, de Jong RJ, Tiren-Verbeet NL, Sommeijer DW, de Heer K, Schaar CG, Sedee RJ, Bosscha K, van den Brekel MW, Petersen JF, Westerman M, Honings J, Takes RP, Houtenbos I, van den Broek WT, de Bree R, Jansen P, Eerenstein SE, Leemans CR, Zijlstra JM, Cuijpers P, van de Poll-Franse LV, Verdonck-de Leeuw IM (2021). Cost-utility of an eHealth application 'Oncokompas' that supports cancer survivors in self-management: results of a randomised controlled trial. J Cancer Surviv.

[220] van der Hout A, van Uden-Kraan CF, Holtmaat K, Jansen F, Lissenberg-Witte BI, Nieuwenhuijzen GAP, Hardillo JA, Baatenburg de Jong RJ, Tiren-Verbeet NL, Sommeijer DW, de Heer K, Schaar CG, Sedee RE, Bosscha K, van den Brekel MW, Petersen JF, Westerman M, Honings J, Takes RP, Houtenbos I, van den Broek WT, de Bree R, Jansen P, Eerenstein SE, Leemans CR, Zijlstra JM, Cuijpers P, van de Poll-Franse LV, Verdonck-de Leeuw IM (2020). Role of eHealth application Oncokompas in supporting self-management of symptoms and health-related quality of life in cancer survivors: a randomised, controlled trial. Lancet Oncol.

[221] van Helmondt SJ, van der Lee ML, van Woezik RAM, Lodder P, de Vries J (2020). No effect of CBT-based online self-help training to reduce fear of cancer recurrence: first results of the CAREST multicenter randomized controlled trial. Psychooncology.

[222] Vogel RI, Niendorf K, Petzel S, Lee H, Teoh D, Blaes AH, Argenta P, Rivard C, Winterhoff B, Lee HY, Geller MA (2019). A patient-centered mobile health application to motivate use of genetic counseling among women with ovarian cancer: a pilot randomized controlled trial. Gynecol Oncol.

[223] Vogel RI, Petzel SV, Cragg J, McClellan M, Chan D, Dickson E, Jacko JA, Sainfort F, Geller MA (2013). Development and pilot of an advance care planning website for women with ovarian cancer: a randomized controlled trial. Gynecol Oncol.

[224] Waller E, Sutton P, Rahman S, Allen J, Saxton J, Aziz O (2022). Prehabilitation with wearables versus standard of care before major abdominal cancer surgery: a randomised controlled pilot study (trial registration: NCT04047524). Surg Endosc.

[225] White V, Farrelly A, Pitcher M, Hill D (2018). Does access to an information-based, breast cancer specific website help to reduce distress in young women with breast cancer? Results from a randomised trial. Eur J Cancer Care (Engl).

[226] Wilkie DJ, Yao Y, Ezenwa MO, Suarez ML, Dyal BW, Gill A, Hipp T, Shea R, Miller J, Frank K, Nardi N, Murray M, Glendenning J, Perez J, Carrasco JD, Shuey D, Angulo V, McCurry T, Martin J, Butler A, Wang ZJ, Molokie RE (2020). A stepped-wedge randomized controlled trial: effects of ehealth interventions for pain control among adults with cancer in hospice. J Pain Symptom Manage.

[227] Willems RA, Bolman CAW, Mesters I, Kanera IM, Beaulen AAJM, Lechner L (2017). Short-term effectiveness of a web-based tailored intervention for cancer survivors on quality of life, anxiety, depression, and fatigue: randomized controlled trial. Psychooncology.

[228] Willems RA, Mesters I, Lechner L, Kanera IM, Bolman CAW (2017). Long-term effectiveness and moderators of a web-based tailored intervention for cancer survivors on social and emotional functioning, depression, and fatigue: randomized controlled trial. J Cancer Surviv.

[229] Wootten AC, Abbott JAM, Meyer D, Chisholm K, Austin DW, Klein B, McCabe M, Murphy DG, Costello AJ (2015). Preliminary results of a randomised controlled trial of an online psychological intervention to reduce distress in men treated for localised prostate cancer. Eur Urol.

[230] Wu LM, Amidi A, Tanenbaum ML, Winkel G, Gordon WA, Hall SJ, Bovbjerg K, Diefenbach MA (2018). Computerized cognitive training in prostate cancer patients on androgen deprivation therapy: a pilot study. Support Care Cancer.

[231] Xia L (2020). The effects of continuous care model of information-based hospital-family integration on colostomy patients: a randomized controlled trial. J Cancer Educ.

[232] Yount SE, Rothrock N, Bass M, Beaumont JL, Pach D, Lad T, Patel J, Corona M, Weiland R, Del Ciello K, Cella D (2014). A randomized trial of weekly symptom telemonitoring in advanced lung cancer. J Pain Symptom Manage.

[233] Yun YH, Lee KS, Kim YW, Park SY, Lee ES, Noh DY, Kim S, Oh JH, Jung SY, Chung K, Lee YJ, Jeong S, Park KJ, Shim YM, Zo JI, Park JW, Kim YA, Shon EJ, Park S (2012). Web-based tailored education program for disease-free cancer survivors with cancer-related fatigue: a randomized controlled trial. J Clin Oncol.

[234] Yun YH, Lim CI, Lee ES, Kim YT, Shin KH, Kim YW, Park KJ, Jeong S, Ryu KW, Han W, Jung KH, Park SC, Kim MS, Kim S, Shim YM, Oh JH, Lee JM, Ryoo S, Woo J, Noh D, Park JW, In Moon B, Kim HJ, Nam SJ, Lee DH, Zo JI, Park SM, Kang E, Rhee Y, Jung JY, Sim JA, Lee J, Shin A (2020). Efficacy of health coaching and a web-based program on physical activity, weight, and distress management among cancer survivors: a multi-centered randomised controlled trial. Psychooncology.

[235] Zachariae R, Amidi A, Damholdt MF, Clausen CDR, Dahlgaard J, Lord H, Thorndike FP, Ritterband LM (2018). Internet-delivered cognitive-behavioral therapy for insomnia in breast cancer survivors: a randomized controlled trial. J Natl Cancer Inst.

[236] Zamorano AS, Wilson EM, Liu J, Leon A, Kuroki LM, Thaker PH, McCourt CK, Fuh KC, Powell MA, Mutch DG, Evanoff BA, Colditz GA, Hagemann AR (2021). Text-message-based behavioral weight loss for endometrial cancer survivors with obesity: a randomized controlled trial. Gynecol Oncol.

[237] Zhang Q, Li F, Zhang H, Yu X, Cong Y (2018). Int J Nurs Stud.

[238] Zhou K, Wang W, Zhao W, Li L, Zhang M, Guo P, Zhou C, Li M, An J, Li J, Li X (2020). Benefits of a WeChat-based multimodal nursing program on early rehabilitation in postoperative women with breast cancer: a clinical randomized controlled trial. Int J Nurs Stud.

[239] Anderson KO, Palos GR, Mendoza TR, Cleeland CS, Liao KP, Fisch MJ, Garcia-Gonzalez A, Rieber AG, Nazario LA, Valero V, Hahn KM, Person CL, Payne R (2015). Automated pain intervention for underserved minority women with breast cancer. Cancer.

[240] Bantum EO, Albright CL, White KK, Berenberg JL, Layi G, Ritter PL, Laurent D, Plant K, Lorig K (2014). Surviving and thriving with cancer using a web-based health behavior change intervention: randomized controlled trial. J Med Internet Res.

[241] Basch E, Deal AM, Dueck AC, Scher HI, Kris MG, Hudis C, Schrag D (2017). Overall survival results of a trial assessing patient-reported outcomes for symptom monitoring during routine cancer treatment. JAMA.

[242] Berg CJ, Vanderpool RC, Getachew B, Payne JB, Johnson MF, Sandridge Y, Bierhoff J, Le L, Johnson R, Weber A, Patterson A, Dorvil S, Mertens A (2020). A hope-based intervention to address disrupted goal pursuits and quality of life among young adult cancer survivors. J Cancer Educ.

[243] Berry DL, Halpenny B, Hong F, Wolpin S, Lober WB, Russell KJ, Ellis WJ, Govindarajulu U, Bosco J, Davison BJ, Bennett G, Terris MK, Barsevick A, Lin DW, Yang CC, Swanson G (2013). The Personal Patient Profile-Prostate decision support for men with localized prostate cancer: a multi-center randomized trial. Urol Oncol.

[244] Choi Yoo SJ, Nyman JA, Cheville AL, Kroenke K (2014). Cost effectiveness of telecare management for pain and depression in patients with cancer: results from a randomized trial. Gen Hosp Psychiatry.

[245] Denis F, Yossi S, Septans AL, Charron A, Voog E, Dupuis O, Ganem G, Pointreau Y, Letellier C (2017). Improving survival in patients treated for a lung cancer using self-evaluated symptoms reported through a web application. Am J Clin Oncol.

[246] Diefenbach MA, Mohamed NE, Butz BP, Bar-Chama N, Stock R, Cesaretti J, Hassan W, Samadi D, Hall SJ (2012). Acceptability and preliminary feasibility of an internet/CD-ROM-based education and decision program for early-stage prostate cancer patients: randomized pilot study. J Med Internet Res.

[247] Ehrbar V, Urech C, Rochlitz C, Zanetti Dällenbach R, Moffat R, Stiller R, Germeyer A, Nawroth F, Dangel A, Findeklee S, Tschudin S (2019). Randomized controlled trial on the effect of an online decision aid for young female cancer patients regarding fertility preservation. Hum Reprod.

[248] Forbes CC, Blanchard CM, Mummery WK, Courneya KS (2015). Feasibility and preliminary efficacy of an online intervention to increase physical activity in nova scotian cancer survivors: a randomized controlled trial. JMIR Cancer.

[249] Gustafson DH, Hawkins R, Pingree S, McTavish F, Arora NK, Mendenhall J, Cella DF, Serlin RC, Apantaku FM, Stewart J, Salner A (2001). Effect of computer support on younger women with breast cancer. J Gen Intern Med.

[250] Hatchett A, Hallam JS, Ford MA (2013). Evaluation of a social cognitive theory-based email intervention designed to influence the physical activity of survivors of breast cancer. Psychooncology.

[251] Kato PM, Cole SW, Bradlyn AS, Pollock BH (2008). A video game improves behavioral outcomes in adolescents and young adults with cancer: a randomized trial. Pediatrics.

[252] Kunin-Batson A, Steele J, Mertens A, Neglia JP (2016). A randomized controlled pilot trial of a web-based resource to improve cancer knowledge in adolescent and young adult survivors of childhood cancer. Psychooncology.

[253] Lee MK, Yun YH, Park H, Lee ES, Jung KH, Noh DY (2014). A web-based self-management exercise and diet intervention for breast cancer survivors: pilot randomized controlled trial. Int J Nurs Stud.

[254] Ruland CM, White T, Stevens M, Fanciullo G, Khilani SM (2003). Effects of a computerized system to support shared decision making in symptom management of cancer patients: preliminary results. J Am Med Inform Assoc.

[255] Valle CG, Deal AM, Tate DF (2017). Preventing weight gain in African American breast cancer survivors using smart scales and activity trackers: a randomized controlled pilot study. J Cancer Surviv.

[256] Viers BR, Lightner DJ, Rivera ME, Tollefson MK, Boorjian SA, Karnes RJ, Thompson RH, O'Neil DA, Hamilton RL, Gardner MR, Bundrick M, Jenkins SM, Pruthi S, Frank I, Gettman MT (2015). Efficiency, satisfaction, and costs for remote video visits following radical prostatectomy: a randomized controlled trial. Eur Urol.

[257] Wheelock AE, Bock MA, Martin EL, Hwang J, Ernest ML, Rugo HS, Esserman LJ, Melisko ME (2015). SIS.NET: a randomized controlled trial evaluating a web-based system for symptom management after treatment of breast cancer. Cancer.

[258] Yanez B, McGinty HL, Mohr DC, Begale MJ, Dahn JR, Flury SC, Perry KT, Penedo FJ (2015). Feasibility, acceptability, and preliminary efficacy of a technology-assisted psychosocial intervention for racially diverse men with advanced prostate cancer. Cancer.

[259] Zernicke KA, Campbell TS, Speca M, McCabe-Ruff K, Flowers S, Carlson LE (2014). A randomized wait-list controlled trial of feasibility and efficacy of an online mindfulness-based cancer recovery program: the eTherapy for cancer applying mindfulness trial. Psychosom Med.

[260] Yanez B, Oswald LB, Baik SH, Buitrago D, Iacobelli F, Perez-Tamayo A, Guitelman J, Penedo FJ, Buscemi J (2020). Brief culturally informed smartphone interventions decrease breast cancer symptom burden among Latina breast cancer survivors. Psychooncology.

[261] Velikova G, Booth L, Smith AB, Brown PM, Lynch P, Brown JM, Selby PJ (2004). Measuring quality of life in routine oncology practice improves communication and patient well-being: a randomized controlled trial. J Clin Oncol.

[262] Hawley ST, Li Y, An LC, Resnicow K, Janz NK, Sabel MS, Ward KC, Fagerlin A, Morrow M, Jagsi R, Hofer TP, Katz SJ (2018). Improving breast cancer surgical treatment decision making: the iCanDecide randomized clinical trial. J Clin Oncol.

[263] PRISMA for Scoping Reviews. PRISMA.

[264] DeSantis CE, Miller KD, Dale W, Mohile SG, Cohen HJ, Leach CR, Goding Sauer A, Jemal A, Siegel RL (2019). Cancer statistics for adults aged 85 years and older, 2019. CA Cancer J Clin.

[265] Pilleron S, Sarfati D, Janssen-Heijnen M, Vignat J, Ferlay J, Bray F, Soerjomataram I (2019). Global cancer incidence in older adults, 2012 and 2035: a population-based study. Int J Cancer.

[266] Howlader N, Noone AM, Krapcho M, Miller D, Brest A, Yu M, Ruhl J, Tatalovich Z, Mariotto A, Lewis DR, Chen HS, Feuer EJ, Cronin KA (2020). SEER Cancer Statistics Review, 1975-2018. National Cancer Institute.

[267] Kemp E, Trigg J, Beatty L, Christensen C, Dhillon HM, Maeder A, Williams PA, Koczwara B (2021). Health literacy, digital health literacy and the implementation of digital health technologies in cancer care: the need for a strategic approach. Health Promot J Austr.

[268] Adam R, McMichael D, Powell D, Murchie P (2019). Publicly available apps for cancer survivors: a scoping review. BMJ Open.

[269] Lambert SD, Harrison JD, Smith E, Bonevski B, Carey M, Lawsin C, Paul C, Girgis A (2012). The unmet needs of partners and caregivers of adults diagnosed with cancer: a systematic review. BMJ Support Palliat Care.

[270] Charalambous A (2019). Utilizing the advances in digital health solutions to manage care in cancer patients. Asia Pac J Oncol Nurs.

[271] Kotronoulas G, Kearney N, Maguire R, Harrow A, Di Domenico D, Croy S, MacGillivray S (2014). What is the value of the routine use of patient-reported outcome measures toward improvement of patient outcomes, processes of care, and health service outcomes in cancer care? A systematic review of controlled trials. J Clin Oncol.

[272] Barbor M (2019). Engaging patients in their cancer care through digital health. Oncology Pharmacist.

