# Supplementary material for: Digital Health Interventions for Adult Patients With Cancer Evaluated in Randomized Controlled Trials: Scoping Review
Source: J Med Internet Res. 2023 Jan 6;25:e38333. doi: 10.2196/38333 (PMC9862347; doi:10.2196/38333)
Supplement: Multimedia Appendix 1 [file jmir_v25i1e38333_app1.pdf]

‘Adult’ AND‘ Patient’ AND ‘Cancer’ AND ‘Digital health’ AND’ Randomized controlled trial.’

(((((Adult[MeSH] OR Adult[TIAB] OR adults[TIAB]) AND (patients[MeSH] OR patients[TIAB] OR patient[TIAB])) OR ((Adult[MeSH] OR Adult[TIAB] OR adults[TIAB]) AND (Cancer survivors[MeSH] OR "cancer survivor"[TIAB] OR "cancer survivors"[TIAB]))) AND ("neoplasms"[MeSH Terms] OR "neoplasms"[TIAB] OR "cancer"[TIAB] OR "cancers"[TIAB] OR "tumor"[TIAB] OR leukemi*[TIAB] OR leukaemi*[TIAB] OR lymphoma*[TIAB] OR myeloma*[TIAB])) AND ((eHealth[TIAB] OR "electronic health"[TIAB] OR digital technology[TIAB] OR "health information technology"[TIAB] OR "health technology"[TIAB] OR ICT[TIAB] OR telemedicine[TIAB] OR telehealth[TIAB] OR telecommunication [TIAB] OR telecommunications [TIAB] OR teleconference [TIAB] OR telecoaching [TIAB] OR mhealth[TIAB] OR m-health[TIAB] OR teleconsult[TIAB] OR teleconsultation[TIAB] OR telecare[TIAB] OR telemonitoring[TIAB] OR Tele-oncology[TIAB] OR "Tele oncology"[TIAB] OR teleoncology[TIAB] OR telenursing[TIAB] OR "computer assisted therapy" [TIAB] OR videoconsult[TIAB] OR "video consultation"[TIAB] OR videocommunication[TIAB] OR "video communication"[TIAB] OR virtual clinic[TIAB] OR "Computer Communication Networks"[TIAB] OR "Electronic communication"[TIAB] OR "communication technology" [TIAB] OR "communications media"[TIAB] OR multimedia[TIAB] OR "educational technology"[TIAB] OR cellphone[TIAB] OR cellphones[TIAB] OR smartphone[TIAB] OR "mobile device"[TIAB] OR wearable [TIAB] OR "handheld computers"[TIAB] OR "computers, handheld"[TIAB] OR "tablet computer"[TIAB] OR "tablet computers"[TIAB] OR iPad[TIAB] OR laptops[TIAB] OR laptop[TIAB] OR digital device [TIAB] OR e-mail[TIAB] OR "electronic mail"[TIAB] OR "mobile application"[TIAB] OR app[TIAB] OR apps[TIAB] OR "text messaging"[TIAB] OR sms[TIAB] OR text[TIAB] OR texts[TIAB] OR mms[TIAB] OR "short messaging service"[TIAB] OR "automated telephone"[TIAB] OR IVR[TIAB] OR "interactive voice response"[TIAB] OR "voice message"[TIAB] OR "voice messages"[TIAB] OR voicemail[TIAB] OR "voice mail"[TIAB] OR "social media"[TIAB] OR "online social networks"[TIAB] OR "social computing"[TIAB] OR "social networking"[TIAB] OR game[TIAB] OR gaming[TIAB] OR video[TIAB] OR internet[TIAB] OR web[TIAB] OR web-based[TIAB] OR website[TIAB] OR digital[TIAB] OR online[TIAB] OR virtual[TIAB] OR cyberspace[TIAB]) OR (digital technology[MeSH Terms] OR "Telemedicine"[Mesh] OR "telecommunications"[MeSH Terms] OR Remote Consultation[MeSH] OR Telenursing[MeSH] OR Internet-Based Intervention[MeSH] OR Computer Communication Networks[MeSH] OR Therapy, Computer-Assisted[MeSH] OR Drug Therapy, Computer-Assisted[MeSH] OR multimedia[MeSH] OR "educational technology"[MeSH Terms] OR "Cell Phone"[MeSH] OR Wearable Electronic Devices[MeSH] OR "Mobile Applications"[Mesh] OR "Text Messaging"[Mesh] OR "social media"[MeSH Terms] OR "internet"[MeSH Terms] OR "smartphone"[MeSH Terms] OR

"computers, handheld"[MeSH Terms] OR multimedia[MeSH]) OR "Electronic Health Records"[TIAB] OR "electronic health record"[TIAB] OR "electronic health records"[TIAB] OR "electronic medical record"[TIAB] OR "electronic medical records"[TIAB] OR "patient health record"[TIAB] OR "patient health records"[TIAB] OR "personal health record"[TIAB] OR "personal health records"[TIAB] OR Electronic Health Records[MeSH] OR Patient Generated Health Data[MeSH] OR Health Records, Personal[MeSH] OR Medical Records Systems, Computerized[MeSH])) AND ((("Randomized Controlled Trial"[Publication Type] OR "trial"[TIAB] OR "Randomized"[TIAB] OR "Randomised"[TIAB] OR (("Randomized"[TIAB] OR "Randomised"[TIAB]) AND ("Study"[TIAB] OR "trial"[TIAB]))) NOT ("animals"[MeSH Terms] NOT "humans"[MeSH Terms])) NOT ((("clinical trial"[Publication Type] OR "clinical trials as topic"[MeSH Terms] OR "clinical trial"[All Fields]) AND ("clinical trials, phase i as topic"[MeSH Terms] OR "phase i as topic clinical trials"[All Fields] OR "phase 1"[All Fields])) OR (("clinical trial"[Publication Type] OR "clinical trials as topic"[MeSH Terms] OR "clinical trial"[All Fields]) AND ("clinical trials, phase ii as topic"[MeSH Terms] OR ("clinical"[All Fields] AND "trials"[All Fields] AND "phase"[All Fields] AND "ii"[All Fields] AND "topic"[All Fields]) OR "phase ii as topic clinical trials"[All Fields] OR "phase 2"[All Fields])) OR (("clinical trial"[Publication Type] OR "clinical trials as topic"[MeSH Terms] OR "clinical trial"[All Fields]) AND ("clinical trials, phase iii as topic"[MeSH Terms] OR ("clinical"[All Fields] AND "trials"[All Fields] AND "phase"[All Fields] AND "iii"[All Fields] AND "topic"[All Fields]) OR "phase iii as topic clinical trials"[All Fields] OR "phase 3"[All Fields])) OR (("clinical trial"[Publication Type] OR "clinical trials as topic"[MeSH Terms] OR "clinical trial"[All Fields]) AND ("clinical trials, phase iv as topic"[MeSH Terms] OR ("clinical"[All Fields] AND "trials"[All Fields] AND "phase"[All Fields] AND "iv"[All Fields] AND "topic"[All Fields]) OR "phase iv as topic clinical trials"[All Fields] OR "phase 4"[All Fields])) OR "phase 1" [TIAB] OR "phase 2" [TIAB] OR "phase 3"[TIAB] OR "phase 4"[TIAB] OR "phase I" [TIAB] OR "phase II" [TIAB] OR "phase III"[TIAB] OR "phase IV"[TIAB] OR "phase 1" [TIAB] OR "phase 2" [TIAB] OR "phase 3"[TIAB] OR "phase 4"[TIAB] OR "protocol"[TIAB] OR "case report"[TIAB] OR "qualitative "[TIAB] OR "qualitative study"[TIAB] OR "qualitative research"[TIAB] OR "descriptive study"[TIAB] OR "cross sectional"[TIAB] OR "cross-sectional"[TIAB] OR "observational study"[TIAB] OR "descriptive correlational study"[TIAB] OR "correlational study"[TIAB] OR "cohort" [TIAB] OR "survey" [TIAB] OR "retrospective" [TIAB] OR "review"[TIAB])) Filters: Humans, English, Korean from 1999/1/1 - 2021/12/31
